# Antibody–drug conjugates for breast cancer: a bibliometric study and clinical trial analysis

**DOI:** 10.1007/s12672-024-01192-w

**Published:** 2024-08-02

**Authors:** Mengjie Xing, Zhiyi Li, Yuwan Cui, Minghua He, Yang Xing, Lei Yang, Ziling Liu, Linzhi Luo, Hong Wang, Rui Guo

**Affiliations:** 1 Department of Clinical Laboratory, First Bethune Hospital of Jilin University, Changchun, China; 2 First Bethune Hospital of Jilin University, Changchun, China; 3 College of Computer Science and Technology, Jilin University, Changchun, China

## Abstract

**Background:**

Breast cancer (BC) remains the most commonly malignancy among women worldwide. Although early-stage BC typically presents with curative possibilities, advanced-stage disease, especially with metastasis, is significantly limited in terms of effective therapeutic interventions, thereby establishing it as the second leading cause of cancer-related deaths in women. Antibody–Drug Conjugates (ADCs) establish a groundbreaking class of anti-neoplastic agents characterized by high specificity and targeting precision. These agents have been significant in reshaping the therapeutic approach to breast cancer, especially those subtypes with overexpression of the Human Epidermal Growth Factor Receptor 2 (HER2). Comprising monoclonal antibodies, cytotoxic payloads, and conjugative linkers, ADCs function by specifically targeting antigens on cancer cells, thereby facilitating the intracellular delivery of the toxic payload. The present investigation endeavors to synthesize existing primary research outcomes through rigorous bibliometric and data analytical approaches, thereby elucidating the current research landscape, delineating research foci, and identifying potential avenues for future innovation.

**Methods:**

For bibliometric analysis, a comprehensive data set comprising 2181 entries related to ADCs in breast cancer was retrieved from the Web of Science Core Collection (WoSCC) spanning the years 1999 to 2023. This data was further filtered from the Science Citation Index Expanded (SCI-Expanded). Analysis software tools such as CiteSpace and VOSviewer were employed for multifaceted analyses such as trends of publications, contributions of countries, and burst analytics. In the dimension of clinical trials, we interrogated databases including ClinicalTrials.gov (https://www.clinicaltrials.gov) and the WHO International Clinical Trials Registry Platform (ICTRP) (https://trialsearch.who.int). A total of 239 clinical trials were initially sourced, among which, 175 were from ClinicalTrials.gov and 64 from ICTRP. After repetitive and correlation-based screening, 119 trials specifically addressing ADC therapeutic strategies in breast cancer were included. Analytical algorithms were executed using Microsoft-based software to evaluate treatment paradigms, emergent research themes, and progress.

**Results:**

Our investigations signify a growing trend of research on ADCs, with consistent advancements in scientific achievements. The analysis revealed that variables such as economic stratification of nations, healthcare investment paradigms, and disease incidence rates serve as significant determinants in shaping research output. Geographically, the United States emerged as the predominant contributor to the research corpus (36.56%), closely followed by China (21.33%). The underpinning of research accomplishments was found to be significantly bolstered by advancements in molecular biology, immunology, and genetic research. Moreover, the advent of nuclear magnetic resonance diagnostic modalities has contributed saliently to the diagnostic and therapeutic management of breast cancer.

**Conclusion:**

Our study provides a comprehensive overview of the ADC research landscape through rigorous bibliometric and clinical trial evaluations. At present, the ADC arena has witnessed the successful development and FDA approval of 14 distinct agents, substantially improving the clinical outcomes for a broad spectrum of oncological patients. Future research imperatives may include the exploration of ADCs targeting mutated oncoproteins, dual-specificity ADCs, combination payload strategies, peptide-drug conjugates (PDCs), and non-internalizing ADC modalities. With sustained academic and clinical focus, the ADC domain is poised for transformative advancements in targeted therapeutics across a variety of malignancies.

## Introduction

Breast cancer is the most prevalent malignancy in women globally, accounting for a substantial number of cancer-related deaths [1, 2], and the leading cause of death in 12 regions of the world [3]. The disease is molecularly heterogeneous, classified into subtypes based on the expression of estrogen receptor (ER), progesterone receptor (PR), and HER2 [2]. Notably, triple-negative breast cancer (TNBC) is characterized by an absence of these receptors and is associated with the poorest clinical outcomes [4]. Treatment paradigms diverge according to these molecular subtypes; whereas hormonotherapy shows efficacy in ER, PR, or HER2-positive BC, TNBC is generally managed with chemotherapy [5]. With immunotherapy developing fast these years, immune checkpoint inhibitors (ICIs) monotherapy and immune-based combinations have been put into clinic for TNBC patients [6]. However, adverse effects such as hypertransaminasemia remain an obstacle for their clinical use and monitoring liver function should be highly recommended [7, 8]. Immune-based combinations have reported to be more effective than monotherapy [6], and such combining strategies such as ICIs plus ladiratuzumab vedotin, an antibody–drug conjugate, have been studied actively [9].

ADCs represent an innovative class of anti-neoplastic agents for their heightened specificity and precision [10]. Comprising a monoclonal antibody, a cytotoxic payload, and a conjugative linker, ADCs facilitate targeted delivery of therapeutics to tumor cells [10]. Initially conceptualized in the 1960s, ADCs garnered Food and Drug Administration (FDA) approval in 2000 following extensive research [11]. They have since been instrumental in reshaping the therapeutic landscape of BC, particularly in targeting HER2-positive subtypes. Present-day ADCs for BC include ado-trastuzumab emtansine (T-DM1, fam-trastuzumab deruxtecan-nxki (T-DXd), and Sacituzumab govitecan-hziy [10]. The main types of ADC for BC and the components were listed according to the targets (Table 1). Despite their therapeutic advantages, challenges such as drug resistance and adverse events, including hepatotoxicity and interstitial lung disease, remain areas of concern [12]. Evolving research aims not only to augment therapeutic efficacy but also to minimize toxicity, with ongoing studies focusing on novel targets and component optimization [12, 13]. ADCs exhibit diversity in several key aspects: (a) target antigen diversity: consistent with scientometric findings, current clinical trials have an emphasis on multiple target antigens, notably HER2, TROP2, HER3, B7-H3, FRα, and GPNMB [10, 11]. Such diversity enhances our understanding of breast cancer's heterogeneous nature and points toward targeted therapies for various subtypes. (b) Choice and functionality of antibodies: the preference for human IgG antibodies in ADCs corroborates their suitability for achieving optimal specificity and minimal immunogenicity [14]. The different subclasses namely, IgG1, IgG2, IgG3, and IgG4—provide nuanced options for pharmacokinetic and pharmacodynamic adjustments. (c) Payload pharmacology: while the cytotoxic payloads are essential for therapeutic efficacy, they must also meet specific pharmacological requirements, such as solubility, stability, and conjugacy [32]. (d) The drug-to-antibody ratio (DAR) remains a crucial indicator for balancing therapeutic potency and metabolic clearance. (e) Linker technologies: Linkers in ADCs are far from ancillary; they are critical in modulating both the stability and release kinetics of cytotoxic payloads [14]. Cleavable linkers provide advantages in selective payload release under specific tumor conditions, whereas non-cleavable linkers require lysosomal degradation for a more controlled release.

**Table 1:** Components of main types of ADC for BC

Antibody target	ADC	Monoclonal antibody	Cytotoxic payload	Conjugative linker
HER2	T-DM1	Trastuzumab	Emtansine (DM1)	Non-cleavable
	DS-8201	Trastuzumab	DXd	Cleavable
	RC48-ADC	Trastuzumab	MMAE	Cleavable
	ARX788	Trastuzumab	AS269	Non-cleavable
	SHR-A1811	Trastuzumab	SHR9265	Cleavable
	SYD985	Trastuzumab	Duocarmycin	Cleavable
TROP2	SG	hRS7 IgG1k	SN-38	Cleavable
	Dato-DXd	hTINA1 IgG1k	DXd	Cleavable
	SKB264	hRS7 IgG1k	T-030	Cleavable
HER3	U3-1402	Patritumumab	Deruxtecan	Cleavable
B7-H3	MGC018	MGA017	Duocarmycin	Cleavable
FRα	MORAb-202	Farletuzumab	Eribulin	Cleavable
gpNMB	CDX-011	Glembatumumab	MMAE	Cleavable

Given the rapid advancements in ADC research, it is imperative to outline the current research landscape and emerging themes. Thus, the present review will employ bibliometric analysis and clinical trial assessments to elucidate the contemporary state and prospective trends in ADC applications in BC.

Bibliometrics is a quantitative methodology that employs analytical software tools such as CiteSpace and VOSviewer for comprehensive literature analysis. This approach identifies key contributors in terms of authors and institutions and elucidates emergent research themes through keyword analysis [15, 16]. Despite the voluminous literature on ADCs in BC, to the best of our knowledge, there has not been a dedicated bibliometric study addressing this area. The present study aims to fill this gap by conducting an exhaustive bibliometric analysis of ADC research in BC. A corpus of 2161 articles was compiled from the Web of Science (WoS) database, aiming to establish existing research patterns and future directions that can guide subsequent investigations.

## Materials and methods

### Bibliometric analysis of scientific literature

A systematic bibliometric assessment was performed utilizing the Web of Science Core Collection (WoSCC). Data were extracted from the Science Citation Index Expanded (SCI-Expanded) for the years spanning 1999 to 2023. The search strategy was guided by search terms acquired from the ClinicalTrials.gov database. Our primary search criteria encompassed two thematic strings: TS1, representing breast cancer nomenclature ("breast cancer" OR "breast tumor" OR "breast neoplasm" OR "breast carcinoma"), and TS2, focused on antibody–drug conjugates ("antibody drug conjugate" OR "antibody drug conjugates" OR ADC). Detailed parameters for the literature search are available in the supplementary materials. The search was restricted to articles and reviews published in English.

To mitigate biases associated with database updates, the search was executed on July 31, 2023. This resulted in the retrieval of 2161 records, which were downloaded in both plain text and tab-separated value formats. The WoSCC database offers annual distribution metrics of both publications and citations. It should be emphasized that the WoSCC is the principal data source utilized in CiteSpace (6.1.R3) and VOSviewer (1.6.19) software.

**Fig. 1:**
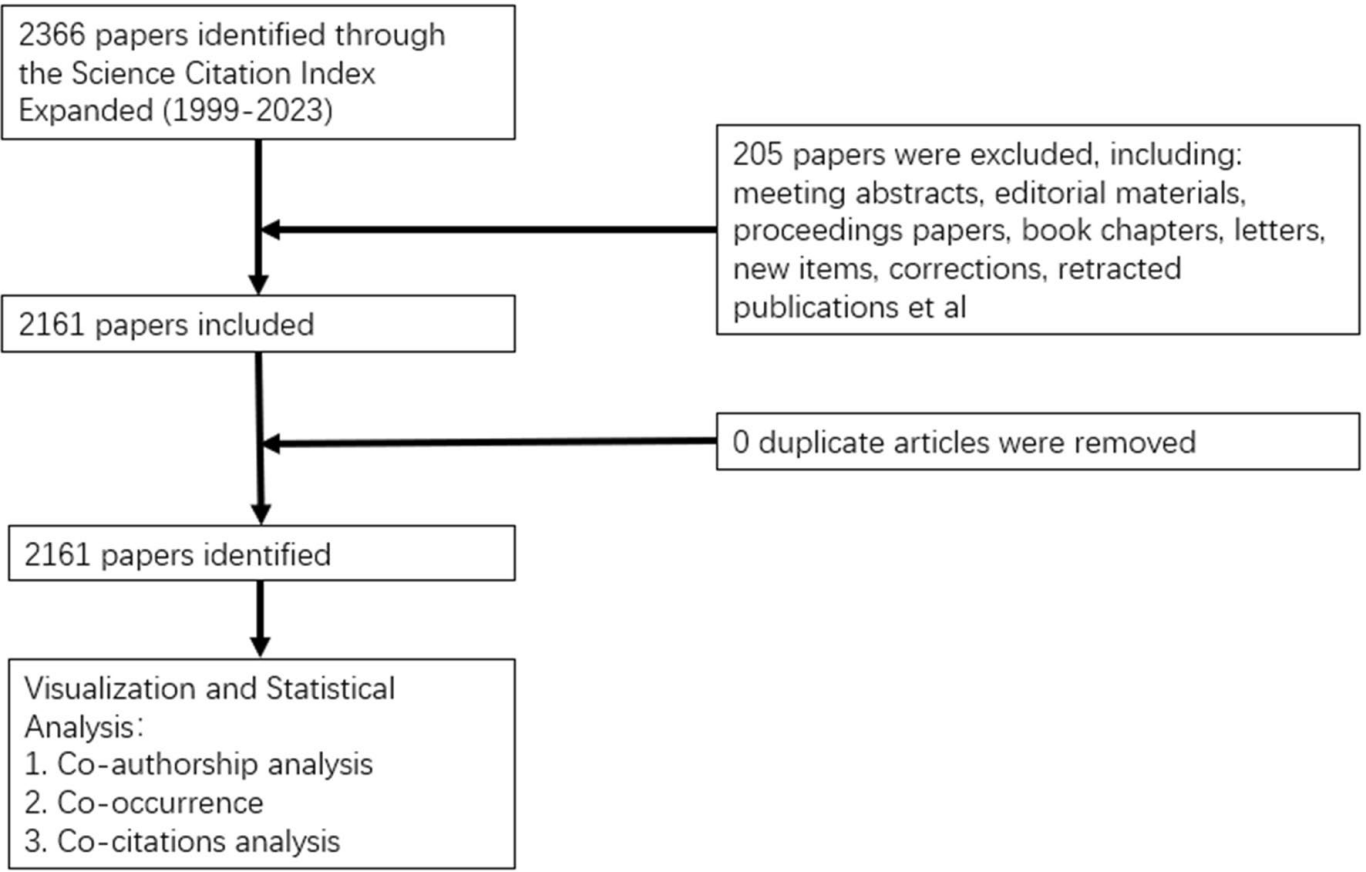
Flowchart of the literature searching and screening in the study

After conducting repetitive and correlation-based screening, the final dataset was analyzed. (Fig. 1) A total of 2705 institutions, 11,958 authors, and 526 journals contributed to these records, representing 69 countries. In addition, 6320 keywords and 3239 author-specific keywords were identified. CiteSpace was configured with specific parameters, including time slices (from January 1999 to July 2023) and pruning techniques, and was utilized to examine variables such as countries, institutions, authors, journals, citations, and keywords.

VOSviewer was employed for analyzing the 2161 records, focusing on geographical and institutional contributions, authors, journals, and emerging research themes.

### Clinical trials analysis

Data were extracted from ClinicalTrials.gov and the World Health Organization's International Clinical Trials Registry Platform (ICTRP). The search parameters were optimized to identify trials focusing on ADC therapies for breast cancer. A total of 175 trials from ClinicalTrials.gov and 64 from ICTRP were retrieved. After conducting repetitive and correlation-based screening, 119 trials were included for analysis. Relevant variables such as treatment modalities, type of ADC, phase, status, and commencement dates were tabulated in Microsoft Excel. Statistical representations were created, focusing primarily on the key targets of ADC therapy.

## Results

### Bibliometric findings

#### Year-wise distribution of publications and citations

The employed search strategy yielded 2161 scholarly articles, which have amassed a cumulative 37,658 citations, excluding 1956 self-citations. When including all citations, the corpus received a total of 66,411 citations, of which 15,715 were self-citations. The mean citation frequency per article was calculated at 38. The h-index was determined to be 127, corroborating the impactful nature and substantial recognition of these scholarly outputs.

#### Temporal trends of publications and citations

**Fig. 2:**
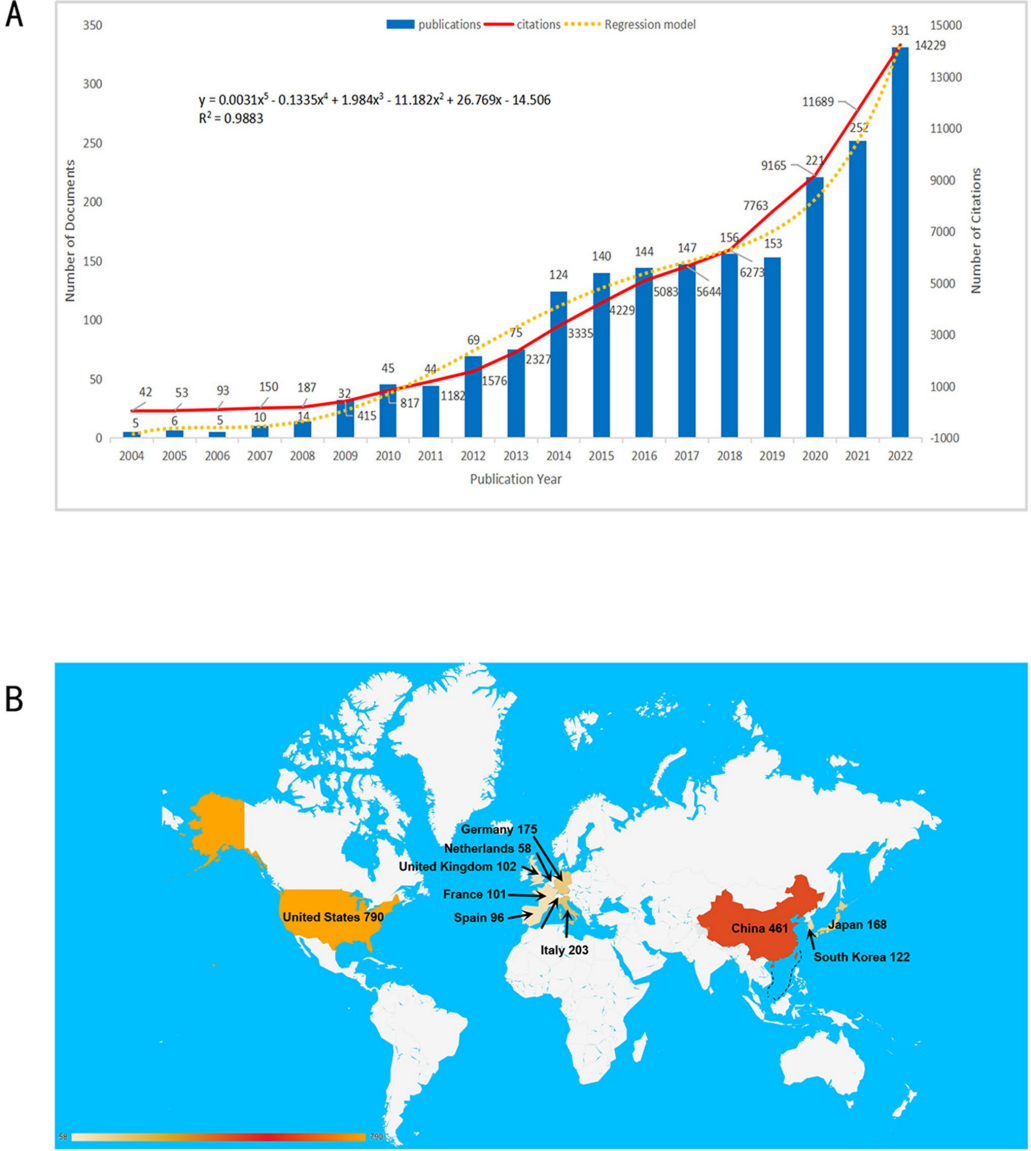
**A** Exponential curve fitting depicting the longitudinal growth trends in publications and citations specific to the realm of antibody–drug conjugates for breast carcinoma. **B** Geographic delineation of research activity, showcasing the top 10 most prolific countries or regions in the arena of antibody–drug conjugates for breast carcinoma (**A** y is the predicted number of publications, and x is the year. R2 represents the regression coefficient, and the closer its value is to 1, the higher the fit degree of the equation. **B** The color change represents the number of published articles, referring to the scale at the bottom left, the number after the country represents the number of breast cancer ADC-related articles published by the country.)

The chronological distribution of both publications and citations from the year 1999 to 2023 were elucidated in Fig. 2A. Microsoft Excel LTSC MSO was deployed for data processing, and a polynomial regression model was established.

#### Geographical disparities in scholarly output

The geographical distribution of academic contributions were illustrated in Fig. 2B. The disparities in scholarly output among nations can potentially be ascribed to a confluence of variables such as economic landscape, investment in healthcare infrastructure, dietary patterns, prevalence of breastfeeding, and mean maternal age at first lactation. These variables warrant a nuanced exploration in future segments of this investigation.

#### Contribution by countries and academic institutions

**Fig. 3:**
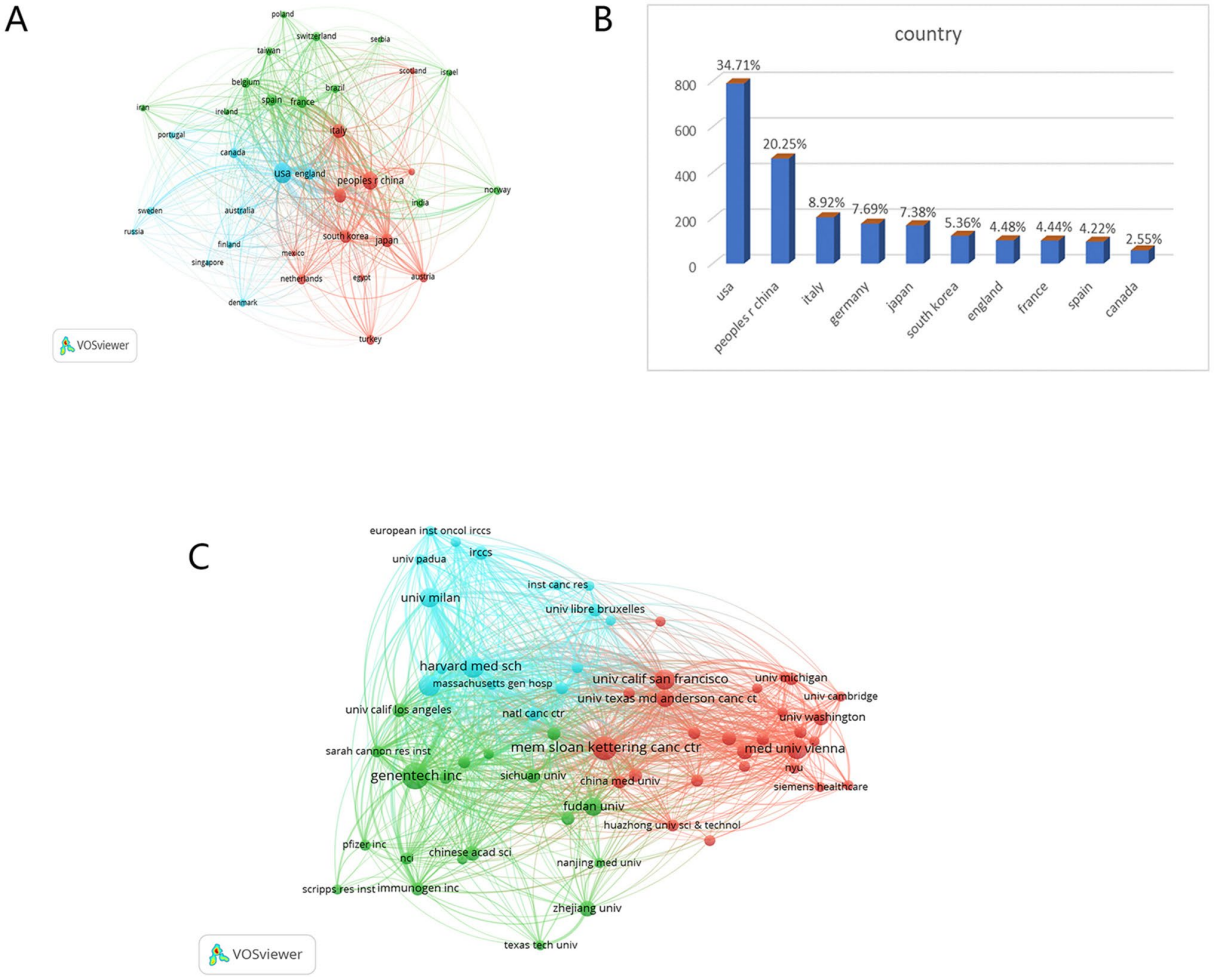
**A** The distribution of countries in terms of publications. **B** A network map showing countries publishing more than 10 papers in this field. **C** A network map showing institutions involved in ADC research in relation to BC (Nodes represent countries and lines represent co-citation relationships.)

A comprehensive VOSviewer assisted analysis was performed to elucidate the geographic dissemination of academic publications in the arena of antibody–drug conjugates targeting breast carcinoma. The analytic output manifested 35 countries or autonomous regions with more than a decennial publication record in this subject area (Fig. 3A). Spearheading this list is the United States, contributing 790 articles and constituting 34.71% of the total academic output. China followed suit with 461 publications, accounting for 20.25% of the total, while Italy was identified as a key contributor with 203 publications, making up 8.92% of the global academic portfolio (Fig. 3B). A methodical inquiry into the institutional contributors was executed using CiteSpace, encapsulating 2705 distinct research entities. Genentech Inc. emerged as a preeminent contributor with a corpus of 71 peer-reviewed articles, thereby leading the field (Fig. 3C). The top decile of publishing institutions comprises five American entities and one Austrian institution (Table 2). These empirical data furnish invaluable insights into the geographical and institutional landscape of research in antibody–drug conjugates targeting breast carcinoma. Notably, the preponderance of scholarly output emanating from the United States accentuates its vital role and substantial contributions in this research area. Within this context, Genentech Inc. stands as a significant correlation for scientific contributions in terms of publication volume.

**Table 2:** The top 10 institutions involved in research on ADC in relation to BC

Rank	Institution	Documents	Rank	Institution	Citations
1	Genentech	71	1	Genentech	9573
2	Memorial Sloan Kettering Cancer Center	60	2	Dana-Farber Cancer Institute	6723
3	Harvard University	54	3	Memorial Sloan Kettering Cancer Center	6239
4	Dana-Farber Cancer Institute	50	4	Seoul National University	4220
5	University of California San Francisco	45	5	Massachusetts Institute of Technology	3285
6	Medical University of Vienna	43	6	Institute Curie	3178
7	University of Milan	41	7	Duke University	3168
8	Fudan University	37	8	Weill Cornell Medicine	2798
9	University of Texas System	33	9	University of Miami	2742
10	University of Washington	28	10	University of California San Francisco	2623

#### Preeminent scholarly contributors

A comprehensive bibliometric assessment executed via VOSviewer revealed a significant community of over 10,000 investigators engaged in research pertaining to antibody–drug conjugates in breast carcinoma. Within this expansive cohort, a subset of 42 authors distinguished themselves by publishing in excess of ten articles in this domain. The author commanding the highest number of scholarly publications is Dr. Katja Pinker, contributing a total of 25 manuscripts. Closely following are Drs. Savannah C. Partridge and Aditya Bardia, each with 23 seminal works, and Dr. Jian Zhou, with 21 publications (Table 3). In terms of scholarly impact, as measured by citation metrics, Dr. Ian E. Krop leads the field with an impressive 5386 citations, succeeded by Dr. guardino, ellie, who has accrued 3468 citations (Table 3).

**Table 3:** The top 10 published authors and co-cited authors involved in research on ADC in relation to BC

Top 10 published authors			Top 10 co-cited authors		
Rank	Author	Counts of documents	Rank	Author	Counts of citations
1	Pinker, Katja	25	1	Krop, Ian E	5386
2	Partridge, Savannah C	23	2	Guardino, Ellie	3468
3	Bardia, Aditya	21	3	Gianni, Luca	3017
4	Curigliano, Giuseppe	21	4	Dieras, Veronique	2976
5	Tolaney, Sara M	19	5	Miles, David	2764
6	Girish, Sandhya	18	6	Welslau, Manfred	2737
7	Cortes, Javier	17	7	Senter, Peter D	2639
8	Helbich, Thomas H	17	8	Girish, Sandhya	2311
9	Kang, Bong Joo	15	9	Modi, Shanu	2281
10	Krop, Ian E	15	10	Sliwkowski, Mark X	1797

#### Academic journal landscape and inter-journal co-citation dynamics

**Fig. 4:**
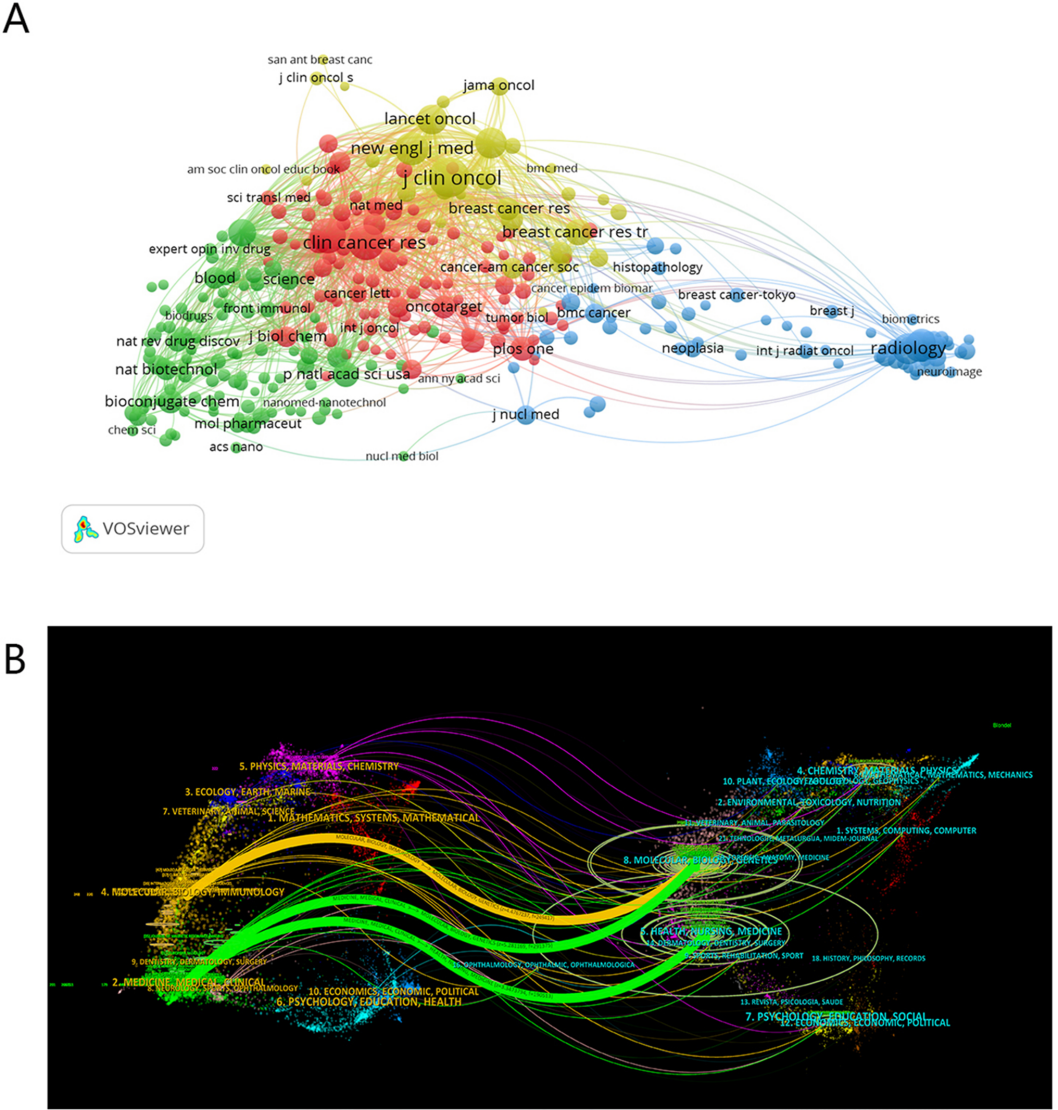
**A** Co-citation analysis of the journals. **B** Overlay graph of journal themes

We identified a total of 526 academic journals contributing to the body of literature surrounding antibody–drug conjugates in breast carcinoma. Among these, the "Journal of Magnetic Resonance Imaging" is most prolific, boasting a cumulative total of 95 articles and registering an impact factor of 4.4 for the year 2022. Other noteworthy contributors include "Cancers," with a total of 80 articles and a 2022 impact factor of 5.2, and "European Radiology," with 61 publications and a 2022 impact factor of 5.9 (Table 4). Notably, Fig. 4A depicts a discernible co-citation network among these journals, suggesting thematic or methodological synergies that warrant further scholarly investigation. It is plausible to hypothesize that these co-citation relationships not only reflect the current multidisciplinary nature of the field but also indicate potential avenues for future integrative research.

**Table 4:** The top 10 published journals involved in research on ADC in relation to BC

Rank	Journal	Counts of articles
1	Journal of magnetic resonance imaging	95
2	Cancers	80
3	European radiology	61
4	Molecular cancer therapeutics	47
5	Frontiers in oncology	45
6	European journal of radiology	42
7	Clinical cancer research	37
8	Magnetic resonance imaging	35
9	Plos one	35
10	Radiology	28

#### Dual-map overlay of journal interdisciplinary communication

The interdisciplinary citation flows between journals through a dual-map overlay was elucidated in Fig. 4B, capturing the thematic intersections within the extant literature on antibody–drug conjugates for breast carcinoma. On the diagram, citing journals are allocated to the left hemisphere, while the cited journals occupy the right hemisphere. Disciplinary labels adjacent to the point of emanation centralize around the centroid of journal clusters corresponding to each academic field. The color-coded trajectories signify various citation pathways, with each pathway's vertical and horizontal ellipses respectively quantifying the volume of published papers and the number of contributing authors for each journal. Remarkably, three principal citation pathways materialized during this analysis: (a) an orange vector signifies a recurrent citation pattern, wherein journals from the domain of Molecular Biology and Genetics are predominantly cited by their counterparts in Molecular Biology and Immunology. (b) A pair of green vectors suggest that journals dedicated to Molecular Biology and Genetics, as well as Health, Nursing, and Medicine, are customarily cited by journals with a Medical or Clinical focus.

#### Leading articles by citation count

The ten articles that have accrued the highest number of citations in the realm of antibody–drug conjugates for breast carcinoma were enumerated in Table 5. Notably, the article entitled "Trastuzumab Emtansine for HER2-Positive Advanced Breast Cancer" stands preeminent with a total of 2396 citations. The salience of this article within the scholarly domain signifies its seminal contributions to the evolving paradigms of antibody–drug conjugate therapy for breast carcinoma. The pronounced citation frequency of this publication corroborates its instrumental role in shaping ongoing inquiries and intellectual dialogues in the field.

**Table 5:** The top 10 literatures involved in ADC research in relation to BC

Rank	Title	Citations	Journal	First author	Publication time
1	Trastuzumab Emtansine for HER2-Positive Advanced Breast Cancer	2396	New England Journal of Medicine	Verma, S	Nov, 2012
2	Antibody therapy of cancer	1512	Nature Reviews Cancer	Scott, AM	Apr, 2012
3	Trastuzumab Emtansine for Residual Invasive HER2-Positive Breast Cancer	1153	New England Journal Of Medicine	von Minckwitz, G	Feb, 2019
4	The ErbB/HER family of protein-tyrosine kinases and cancer	833	Pharmacological Research	Roskoski, R	Jan, 2014
5	Trastuzumab Deruxtecan in Previously Treated HER2-Positive Breast Cancer	804	New England Journal Of Medicine	Modi, S	Feb, 2020
6	Conjugation site modulates the in vivo stability and therapeutic activity of antibody–drug conjugates	698	Nature Biotechnology	Shen, BQ	Feb, 2012
7	Antibody–Drug Conjugates in Cancer Therapy	546	Annual Review Of Medicine	Sievers, EL	Jan, 2013

#### Analysis of keyword co-occurrence

**Fig. 5:**
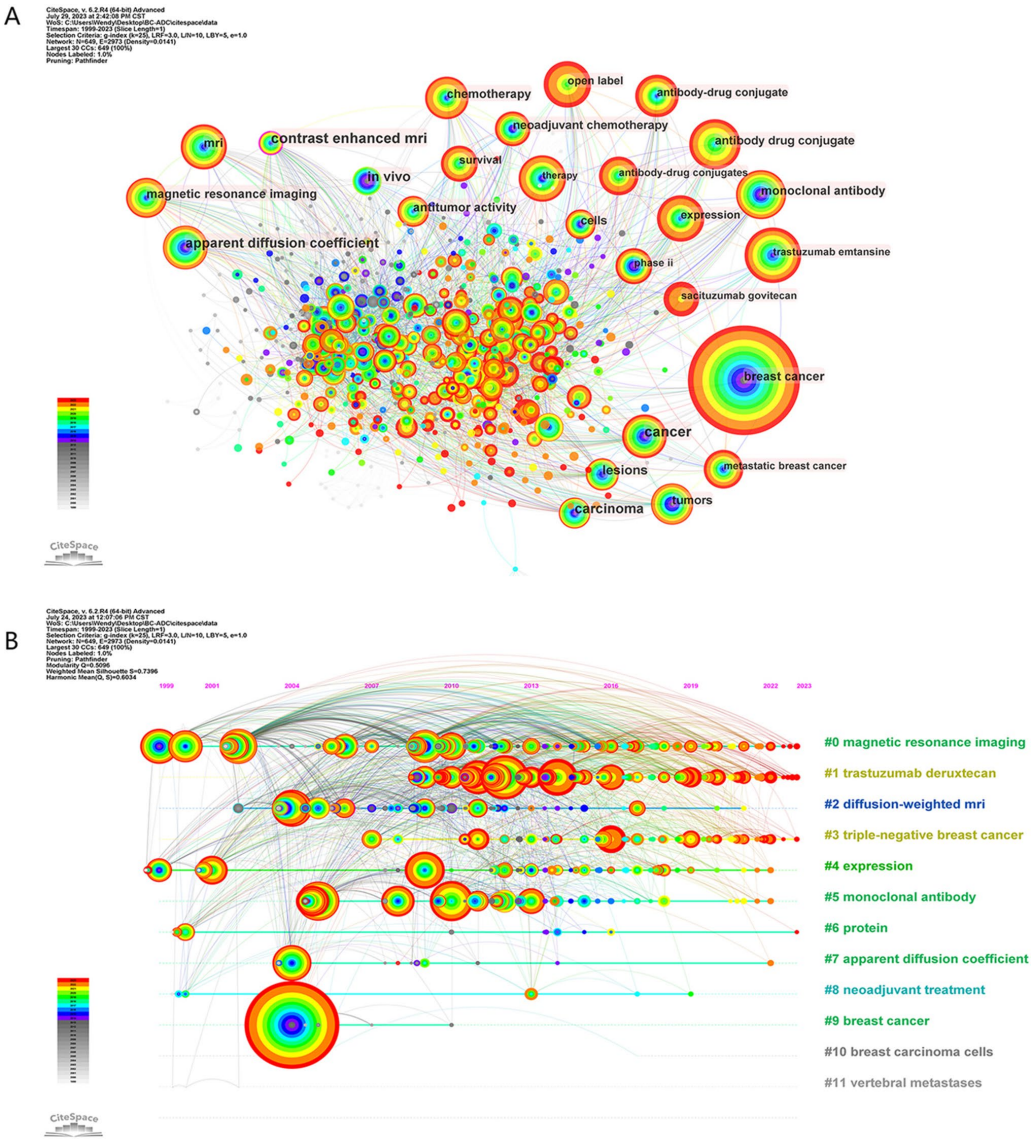
**A** Keywords co-occurrence map involved in ADC research in relation to BC. **B** Cluster analysis of keywords involved in ADC research in relation to BC

Utilizing CiteSpace, we executed a keyword co-occurrence analysis to construct visual maps that delineate the interrelationships among pertinent keywords. This methodological approach efficaciously distills the conceptual framework and loci of intensified research activity within a designated field, thereby furnishing insights into prospective avenues for innovative research (Fig. 5A). The keywords discerned in the context of our investigation are compartmentalized into four distinct categories. Via the co-occurrence analysis of these identified keywords, we are afforded an augmented understanding of the principal thematic underpinnings, relational frameworks, and incipient research trajectories pertinent to the domain of antibody–drug conjugate therapeutics in the context of breast carcinoma. The focus on the interactive dimensions among these elements serves to accentuate their intricate dynamism in the pathogenesis and therapeutic approaches to breast cancer.

In the current study, we utilized CiteSpace to conduct a keyword clustering analysis focusing on terms related to antibody–drug conjugate therapy in the context of breast carcinoma. The number of resultant clusters was ascertained in accordance with the dimensions of each individual cluster, the most extensive of which was denoted as Cluster #0. The analysis yielded a total of 12 distinct clusters. Subsequently, a timeline visualization was constructed via CiteSpace to facilitate a temporal analysis of these clusters. The enumerated clusters encompass: #0 Magnetic Resonance Imaging, #1 Trastuzumab Deruxtecan, #2 Diffusion-Weighted MRI, #3 Triple-Negative Breast Cancer, #4 Expression, #5 Monoclonal Antibody, #6 Protein, #7 Apparent Diffusion Coefficient, #8 Neoadjuvant Treatment, #9 Breast Cancer, #10 Breast Carcinoma Cells, and #11 Vertebral Metastases. This timeline visualization is graphically represented in Fig. 5B.

In terms of research hotspots and developmental trajectories, the thematic categories align with those identified in the prior co-occurrence analysis. Specifically, they are compartmentalized into four categories: (1) Imaging Modalities (#0, #2), (2) Mechanisms Underlying Breast Cancer (#4, #5, #6, #7), (3) Breast Cancer Pathophysiology (#3, #9, #10, #11), and (4) Therapeutic Approaches (#1, #8) (Fig. 8). Additionally, the analysis has disclosed an augmented scholarly interest in clusters #0, #1, and #3 in recent chronological intervals. This intimates that diagnostic imaging modalities, particularly magnetic resonance imaging, have gained substantive prominence in contemporary breast carcinoma research.

#### Analysis of keyword bursts

The utility of keyword burst analysis, executed via CiteSpace, resides in its capacity to pinpoint emergent and swiftly proliferating keywords, hereafter referred to as "burst keywords". In the course of our burst keyword analysis, we selected the 25 most prominent keywords from an expansive set of 6320 keywords in the context of breast cancer-ADCs, thereby illuminating prevailing research foci. The stratification of these keywords was performed on the basis of their burst duration, initiation time, and burst strength metrics. A green linear demarcation within the graphical representation delineates the chronological expanse from 1999 to 2023, whereas a red linear demarcation signifies the temporal duration attributed to each individual burst keyword.

**Fig. 6:**
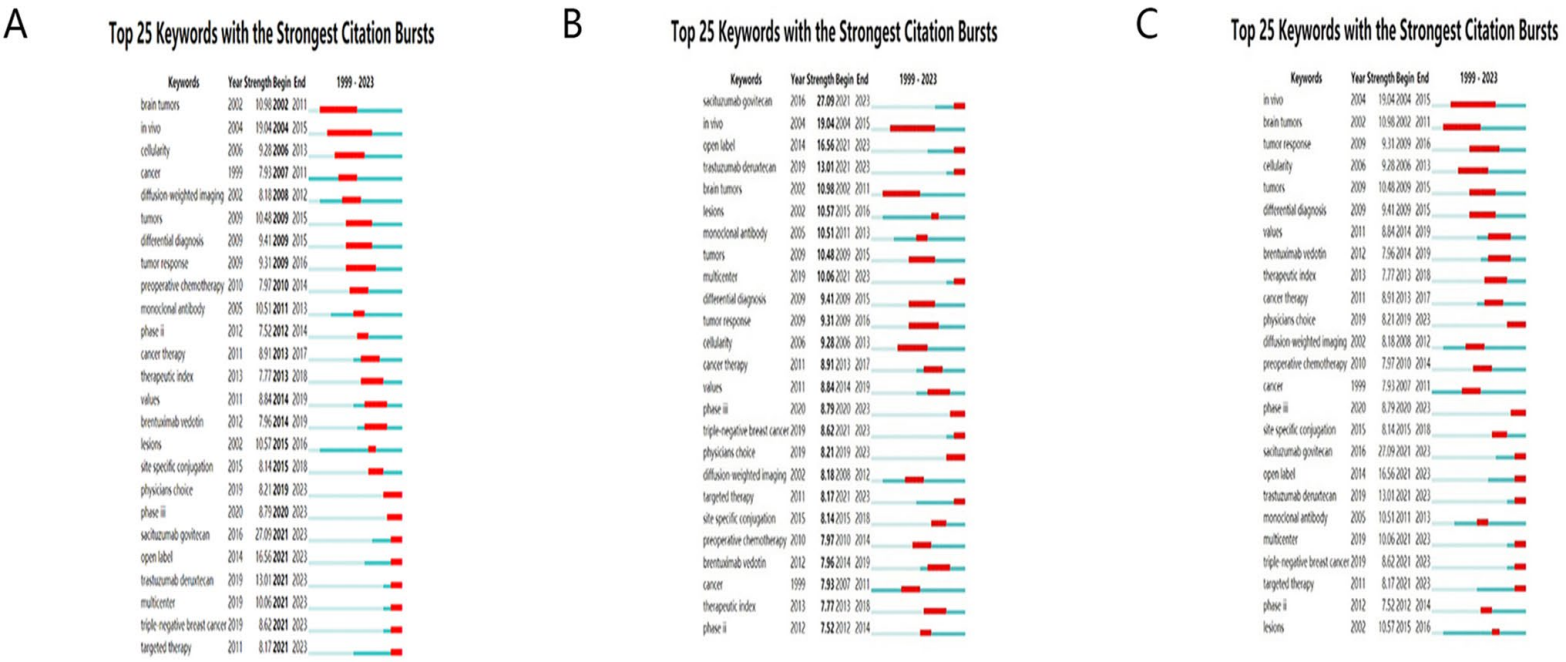
Burst keywords involved in ADC research in relation to BC. **A** Ranking by beginning. **B** Ranking by strengths. **C** Ranking by duration

The keyword "brain tumors" emerged as the inaugural burst keyword in the year 2002, subsequently followed by "in vivo" and "cellularity" in the temporal sequence (Fig. 6A). The keyword "sacituzumab govitecan" manifested the most pronounced burst strength, as illustrated in Fig. 6B. Sacituzumab govitecan represents a therapeutic agent of burgeoning prominence, eliciting substantial scholarly and clinical interest. Within the context of clinical investigations, this pharmaceutical compound has exhibited considerable therapeutic efficacy in the management of certain forms of advanced breast cancer as well as small cell lung cancer, thereby catalyzing heightened consideration of its utility as a treatment modality.

The keyword "in vivo" is conspicuous for its extended duration of bursting activity, as delineated in Fig. 6C. This prolonged burst duration proves a sustained scholarly preoccupation with in vivo methodologies across the span of the analytical timeframe. Such a persistent focus underscores the enduring salience and relativity of in vivo investigative paradigms within the domain of antibody–drug conjugate therapeutics specifically targeting breast carcinoma.

The present analysis attests to the considerable maturation and refinement that the field of antibody–drug conjugates for breast cancer has undergone over the preceding two decades. The geographical dispersion of bursting keywords in initial years may reflect a period of widespread exploratory research, whereas the recent coalescence of bursting keywords suggests an emergent research consensus.

### Analysis of clinical trials

#### Evolution and current landscape of ADC clinical trials

**Fig. 7:**
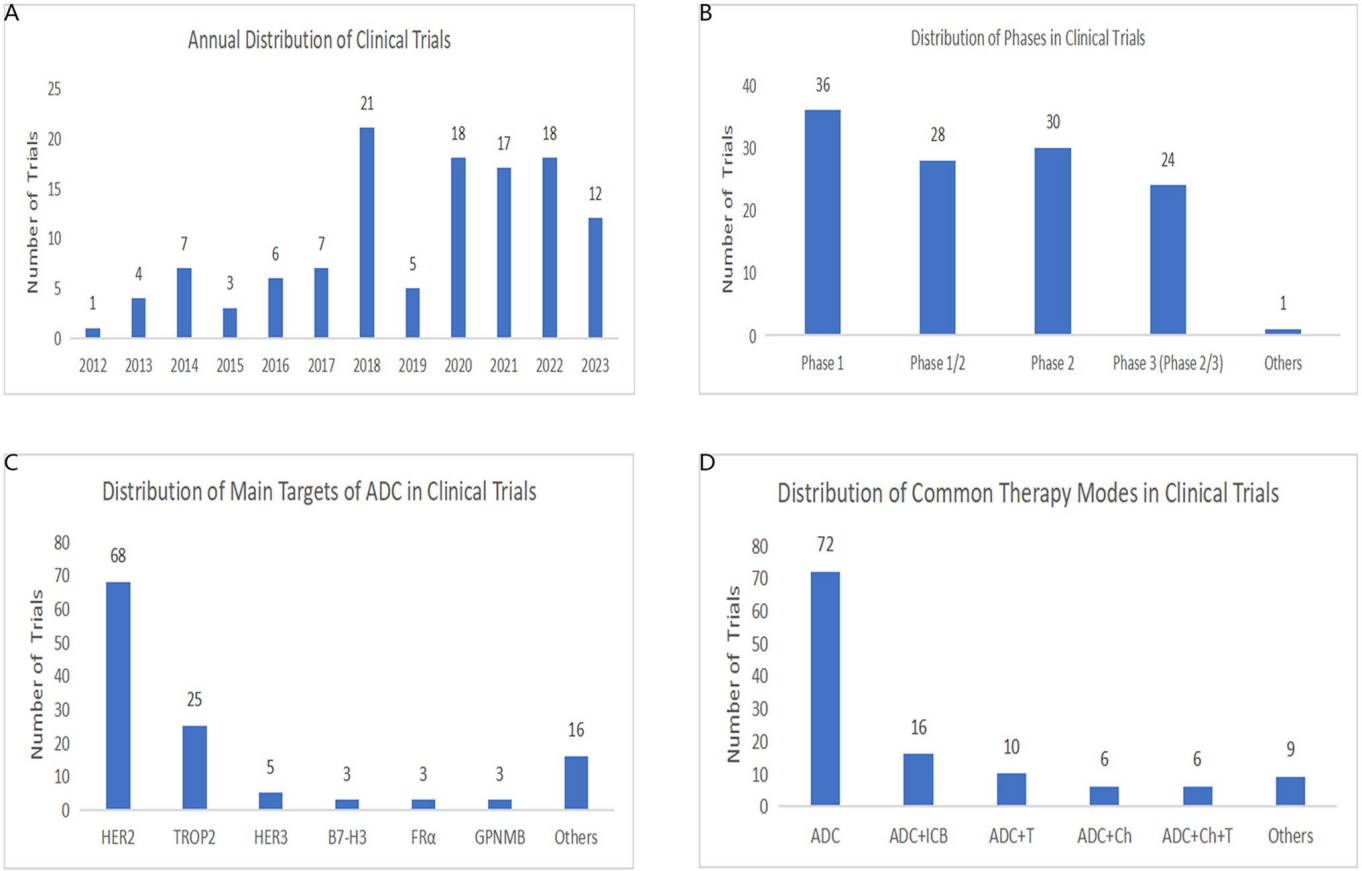
**A** The annual distribution of clinical trials from 2012 to 2023. The start date was regarded as the time of clinical trials. **B** The distribution of phases for all clinical trials. Others refer to the trials without known phases. **C** The distribution of main targets of ADC for all clinical trials. **D** The distribution of common therapy modes for all clinical trials. ADC plus ICB referred to ADC combined with immune checkpoint blocker. (ADC plus T referred to ADC combined with molecular targeting therapy. ADC plus Ch referred to ADC combined with chemotherapy.)

The first clinical trial for ADC was initiated in 2012. Specifically, this was a Phase 1/2 trial designed to assess the safety and tolerability profile of IMMU-132 in epithelial cancers. The corpus of ADC clinical trials has expanded since that juncture, as depicted in Fig. 7A. Although the proliferation of trials was modest in the ensuing five years, a significant crescendo was observed in 2018, reaching an apex of 21 trials. Following a transient decrement in 2019, the volume of trials has stabilized from 2020 through 2022. These data serve as an indicator of the sustained evolution and prospective expansion of ADC clinical trials. Of the 119 enumerated clinical trials, Phase 1 trials comprise the predominant category, totaling 36 instances, succeeded by Phase 2 trials, which account for 30 instances (Fig. 7B). It is of note that Phase 3 trials, enumerated in Table 2, are predominantly characterized by HER2-ADC and TROP2-ADC, signifying that these traditional target loci continue to occupy the vanguard of ADC research and development (Fig. 7C). Subsequent to the advent of the initial TROP2-ADC trial in 2012, HER2 has consistently remained the most extensively investigated target, with trials being executed annually. Concurrently, emergent targets, including Glycoprotein Nonmetastatic Melanoma B (GPNMB), HER3, B7-H3, and Folate Receptor Alpha (FRα), have been introduced in recent years, thereby broadening the therapeutic horizon for ADCs. Despite some of these nascent targets culminating in inconclusive outcomes, they manifest an ongoing dynamic expansion within this research purview. Multiple therapeutic modalities have been explored within the context of ADC clinical trials, encompassing monotherapy with ADC as well as combination strategies (Fig. 7D). A preponderance of trials employs ADC as an isolated therapeutic agent (n = 72), whereas the amalgamation of ADC with immune checkpoint blockers (ICBs) represents another frequently adopted regimen (n = 16). Complementary approaches integrating chemotherapy and targeted therapeutic agents have also been examined as adjuncts to ADC-based treatments.

#### Examination of predominant ADC types in clinical trials

**Fig. 8:**
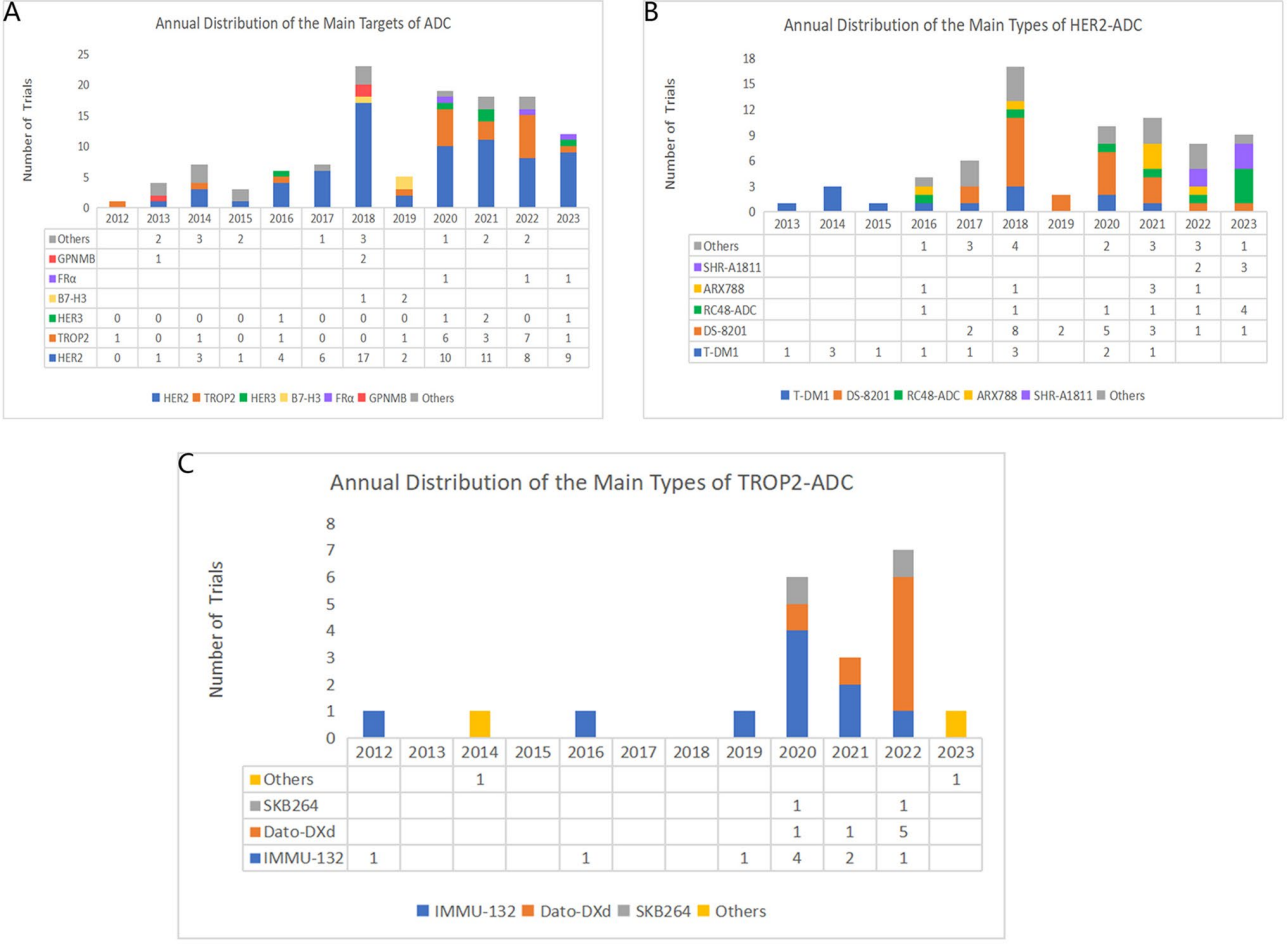
**A** The annual distribution of the main targets of ADC (including HER2, TROP2, HER3, B7-H3, FR a, GPNMB from 2012 to 2023). **B** The annual distribution of the main types of HER2-ADC for all clinical trials. **C** The annual distribution of the main types of TROP2-ADC for all clinical trials

The analysis extends to a nuanced evaluation of ADCs stratified according to distinct targeting moieties (Fig. 8A)**.** While some of these ADCs have secured regulatory approval and are presently deployed in clinical settings, others remain in investigational phases. T-DM1, the inaugural ADC to gain approval for the treatment of breast carcinoma in 2013, has sustained its position as a focal point of scientific inquiry in ensuing years, with 13 trials to its credit. Notably, DS-8201, a HER2-targeted ADC, leads the ensemble with the highest number of clinical trials (n = 22), signaling its latent therapeutic potential and the prospects for expansion into additional clinical indications. Other HER2-specific ADCs, including RC48-ADC, ARX788, and SHR-A1811, have garnered considerable attention in recent academic discourse (Fig. 8B). In the context of TROP2-targeted ADCs, sacituzumab govitecan (IMMU-132) and datopotamab deruxtecan (Dato-DXd) emerge as the most extensively scrutinized candidates, as evidenced in Fig. 8C**.** Of particular significance is the fact that five out of the seven clinical trials pertaining to Dato-DXd are categorized as Phase 3 studies. A comprehensive list of Phase 3 clinical trials is presented in Table 6.

**Table 6:** All the ADC related clinical trials for breast cancer in Phase3 (or Phase2/3)

No	Trial ID	Cancer stage	Cancer feature	Start date	Status	Title
1	NCT04924699	LA/M	HER2+	Jun, 2021	Recruiting	A Study of MRG002 in the Treatment of Patients With HER2-positive Unresectable Locally Advanced or Metastatic Breast Cancer
2	NCT05426486		HER2+	May, 2022	Recruiting	A Study of ARX788 Combined With Pyrotinib Maleate Versus TCBHP (Trastuzumab Plus Pertuzumab With Docetaxel and Carboplatin) as Neoadjuvant Treatment in HER2-positive Breast Cancer Patients
3	NCT03500380	M	HER2+	Apr, 2018	Recruiting	A Study of RC48-ADC Administered Intravenously to Patients With HER2-Positive Metastatic Breast Cancer With or Without Liver Metastases
4	NCT03262935 (EUCTR2017-001994-18-ES)	LA/M	HER2+	Nov, 2017	Has Result	SYD985 vs. Physician's Choice in Participants With HER2-positive Locally Advanced or Metastatic Breast Cancer
5	NCT03529110 (JPRN-JapicCTI-183976)	U/M	HER2+	Aug, 2018	Has Result	DS-8201a Versus T-DM1 for Human Epidermal Growth Factor Receptor 2 (HER2)-Positive, Unresectable and/or Metastatic Breast Cancer Previously Treated With Trastuzumab and Taxane [DESTINY-Breast03]
6	NCT03734029 (JPRN-JapicCTI-184223)	U/M	HER2-low	Dec, 2018	Has Result	Trastuzumab Deruxtecan (DS-8201a) Versus Investigator's Choice for HER2-low Breast Cancer That Has Spread or Cannot be Surgically Removed [DESTINY-Breast04]
7	NCT04494425	M	HER2-low	Jul, 2020	Active, not recruiting	Study of Trastuzumab Deruxtecan (T-DXd) vs Investigator's Choice Chemotherapy in HER2-low, Hormone Receptor Positive, Metastatic Breast Cancer
8	NCT05950945	U/M	HER2-low	Oct, 2023	Not yet recruiting	Trastuzumab Deruxtecan (T-DXd) in Patients Who Have Hormone Receptor-negative and Hormone Receptor-positive HER2-low or HER2 IHC 0 Metastatic Breast Cancer
9	NCT05904964	M	HER2-low	Jul, 2023	Recruiting	Disitamab Vedotin (RC48) in Hormone Receptor Positive, HER2-low Metastatic Breast Cancer (the Rosy Trial)
10	NCT04400695	LA/M	HER2-low	Sep, 2020	Recruiting	A Study of RC48-ADC for the Treatment of Locally Advanced or Metastatic Breast Cancer With Low Expression of HER2
11	NCT03523585 (JPRN-JapicCTI-184017)	U/M	HER2+	Aug, 2018	Has Result	DS-8201a in Pre-treated HER2 Breast Cancer That Cannot be Surgically Removed or Has Spread [DESTINY-Breast02]
12	NCT05104866	U/M	HER2−, HR+	Oct, 2021	Active, not recruiting	A Phase-3, Open-Label, Randomized Study of Dato-DXd Versus Investigator's Choice of Chemotherapy (ICC) in Participants With Inoperable or Metastatic HR-Positive, HER2-Negative Breast Cancer Who Have Been Treated With One or Two Prior Lines of Systemic Chemotherapy (TROPION-Breast01)
13	EUCTR2020-005620-12-ES	U/M	HER2−, HR+	Jan, 2022	Other	A Study of Dato-DXd Versus Investigator's Choice Chemotherapy in Inoperable or Metastatic Hormone Receptor-positive, HER2-negative Breast Cancer
14	NCT05374512 (EUCTR2021-005223-21-DE)	U/M	TNBC	May, 2022	Recruiting	A Study of Dato-DXd Versus Investigator's Choice Chemotherapy in Patients With Locally Recurrent Inoperable or Metastatic Triple-negative Breast Cancer, Who Are Not Candidates for PD-1/PD-L1 Inhibitor Therapy (TROPION-Breast02)
15	NCT04639986	M	HER2−/HR+	Nov, 2020	Active, not recruiting	Asian Study of Sacituzumab Govitecan (IMMU-132) in HR+/HER2− Metastatic Breast Cancer (MBC)
16	NCT05629585		TNBC	Nov, 2022	Recruiting	A Study of Dato-DXd With or Without Durvalumab Versus Investigator's Choice of Therapy in Patients With Stage I-III Triple-negative Breast Cancer Without Pathological Complete Response Following Neoadjuvant Therapy (TROPION-Breast03)
17	NCT04595565 (EUCTR2019-004100-35-DE, EUCTR2019-004100-35-ES)	P	HER-	Oct, 2020	Recruiting	Sacituzumab Govitecan in Primary HER2-negative Breast Cancer

## Discussion

### Insights from bibliometric analysis

Bibliometric analysis serves as a quantitative and qualitative tool for systematically characterizing the landscape of scientific literature. By tracing the nodes and connections within the intricate web of scholarly contributions, bibliometrics offers a holistic yet granular view of the field.

*Current state of research in breast cancer antibody–drug conjugates* The present study substantiates the expansive research on ADCs for breast cancer. The time span under review (1999–2023) saw the emergence of 2161 papers spanning reviews and original research, hailing from 67 countries and involving 2705 institutions and 11,958 individual contributors. These contributions have been featured in a remarkable 526 distinct journals, amassing a plethora of 6320 keywords and 3239 author-specific keywords. This volume of activity has culled a total of 37,658 citations and 66,411 references. Significantly, an h-index of 127 confirms the broad academic acknowledgment of this body of work.

*Anticipated trajectory of breast cancer ADC research* The evidence provided within this study obviates the need for further elaboration regarding the promising prospects of ADCs in breast cancer. Our examination of publication trends, harnessed through statistical models—ranging from linear and exponential functions to higher-order polynomial equations—provides quantitative substantiation of this optimism. The fifth-degree polynomial model, characterized by an R-squared value of 0.9883, was deemed the most representative fit. Such a high coefficient of determination suggests that nearly 99% of the variability in the data can be accounted for by this model. Our forecasts project an escalation in the number of publications to approximately 208.09, 243.86, and 281.45 for the years 2023, 2024, and 2025, respectively. By acknowledging these caveats, we intend to equip researchers and stakeholders with a nuanced understanding of the state and progress of ADC research in breast cancer, thereby facilitating informed decisions for future scientific investigations.

*Geographical distribution of contributions in breast cancer ADC research* Our bibliometric analysis delineates a geography of intellectual contributions with the United States, China, Italy, Germany, Japan, South Korea, the United Kingdom, France, Spain, and the Netherlands at the forefront of ADC research in breast cancer. The network diagrams illustrated in Fig. 3A depict the interconnectedness of these nations, manifesting their roles as thought leaders and collaborators in the field. Several country-specific factors contribute to the landscape of breast cancer treatment needs. These primarily include disease incidence rates and the level of economic development. High-income countries often invest more heavily in healthcare due to public expectation for advanced medical resources, thus necessitating greater treatment demand. In summary, the geographical differences in ADC research and treatment needs for breast cancer are influenced by a complex of socio-economic, demographic, and institutional factors.

*Countries and institution contributions in breast cancer ADC research* The economic affluence, often measured through GDP, manifests as a critical determinant in shaping the country's scientific output in breast cancer ADC research. This trend implies that countries with robust economies are often the ones that can allocate more resources toward scientific endeavors. Moreover, a robust economy allows for more extensive healthcare coverage, thereby fostering an environment that is conducive to clinical trials and drug development, key components of translational research in the realm of antibody–drug conjugates [17] (Table 7). Several factors influence countries' and institutions' investments in ADC research for breast cancer, including: research infrastructure, funding mechanisms, medical facilities, synergistic collaboration and multidisciplinarity, and a balance between commercial interests and academic pursuits, as well as institutional policies. In summary, this analysis provides a window into the intricate interplay of diverse factors, revealing the underlying dynamics that unite various entities into a unified and vibrant research community.

**Table 7:** Comparison of countries with economic level (GDP/2022) and number of publications in ADC for breast cancer (top 10 publications are marked in bold)

Rank by GDP	Country	GDP (billion dollars)	Rank by publication
1	**United States**	25,035.16	1
2	**China**	18,321.20	2
3	**Japan**	4300.62	5
4	**Germany**	4031.15	4
5	India	3468.57	
6	**United Kingdom**	3198.47	7
7	**France**	2778.09	8
8	Canada	2200.35	
9	Russia	2133.09	
10	**Italy**	1996.93	3
11	Islamic Republic of Iran	1973.74	
12	Brazil	1894.71	
13	**Korea**	1734.21	6
14	Australia	1724.79	
15	Mexico	1424.53	
16	**Spain**	1389.93	9
17	Indonesia	1289.43	
18	Saudi Arabia	1010.59	
19	**Netherlands**	990.583	10
20	Turkey	853.487	

*Keyword analysis and research hotspots* The timeline of research directions and the evolution of treatment areas are evident from the analysis of keywords. In the early stages, research primarily focused on imaging diagnosis represented by "#0 magnetic resonance imaging" and "#2 diffusion-weighted MRI," as well as exploring breast cancer pathological mechanisms with "#4 expression" and "#9 breast cancer". After 2007, "#5 monoclonal antibodies" indicated continued enthusiasm for breast cancer research entering the molecular field. "#1 pertuzumab" also gradually gained attention, heralding the era of "targeted therapy" for breast cancer treatment. Pertuzumab is a monoclonal antibody targeting HER2 and is mainly used to treat HER2-overexpressing breast cancer. HER2 overexpression leads to excessive binding with human epidermal growth factor (EGF), resulting in excessive intracellular signaling and rapid cell growth. Pertuzumab works by binding to HER2 receptors, thereby blocking this process and inhibiting cancer cell growth [18]. Applying CiteSpace to keyword analysis, we examined the bursts of keywords in terms of their start time, burst strength, and duration. By omitting ambiguous terms like "brain tumors," "cancer," and "tumors," we focused on the early and clearly directed keywords like "in vivo," "cellularity," and "diffusion-weighted imaging." This reveals that early research directions primarily focused on in vivo studies, cellular mechanisms, and advancements in imaging diagnostics. The burst keywords with the highest ranks include "Sacituzumab govitecan," "in vivo," "open label," and "trastuzumab deruxtecan." "Sacituzumab govitecan" is the first TROP-2 targeted monoclonal antibody/ADC drug, combining humanized monoclonal antibodies targeting Trop-2 and SN-38. This drug can deliver high concentrations of SN-38 to tumors, filling a gap in the treatment of (TNBC [19]. TNBC lacks ER, PR, and HER2 expression, constituting about 15% of invasive breast cancers, known for its aggressive nature and poor prognosis [20, 21]. "Open label" reveals the specifics of ongoing treatments and interventions, which, although lacking blinding, is a necessary approach for ethical and practical reasons in clinical research. However, this approach has limitations and biases that need to be addressed when interpreting the results [22, 23].

*Literature analysis* Co-citation analysis is a bibliometric method used to identify relationships between various scientific publications within a research field. When two or more articles simultaneously cite a preceding article, a co-citation relationship is established. The frequency of co-citation for an article often indicates its influence in the field. High levels of co-citation suggest that a paper is frequently referenced in subsequent research, signifying its important role in shaping the knowledge and understanding of the field. As shown in the table, the co-cited articles primarily come from high-quality journals, including New England Journal of Medicine, Nature Reviews Cancer, and Nature Biotechnology. These articles trace the development of breast cancer diagnosis and the evolution of targeted therapies.

By examining the top ten co-cited articles within a specific field, we can gain an overview of the most influential ideas, research, and discoveries. These highly co-cited articles often constitute the "core" literature or "cornerstones" of the field, as they are frequently referenced in different studies due to their pioneering or foundational nature. This analysis not only helps to understand the main contributions and trends within the field but also provides insights into its evolution and direction.

### Clinical trial analysis of ADCs

The clinical trial data substantiate and elaborate on the scientometric assessments, offering a nuanced understanding of both therapeutic opportunities and challenges in the realm of breast cancer ADC research. This multidimensional analysis forms a cornerstone for prospective research and clinical applications in this dynamically progressing field.

#### Monotherapy with antibody–drug conjugates

##### HER2-Targeted ADCs

HER2, a transmembrane growth factor receptor, is ubiquitously expressed across a spectrum of normal adult tissues [24]. Furthermore, it serves as both a biomarker and a therapeutic target in the context of malignancy. HER2-positive breast cancer is often associated with an adverse prognostic landscape owing to elevated cellular proliferation rates. However, it is noteworthy that this subtype generally exhibits heightened sensitivity to cytotoxic interventions. The monoclonal antibody trastuzumab has gained substantial attention as an effective HER2 inhibitor [13]. In 2013, the FDA conferred approval upon T-DM1, marking it as the inaugural HER2-targeted ADC indicated for the management of breast cancer [10]. Since this regulatory milestone, clinical trials exploring the efficacy and safety of HER2-targeted ADCs have proliferated.

*Trastuzumab emtansine (T-DM1): pioneering the HER2-targeted ADC landscape:* T-DM1, the trailblazing HER2-targeted ADC, comprises the monoclonal antibody trastuzumab, the cytotoxic payload emtansine (DM1), and a non-cleavable linker [25, 26]. In a landmark development, T-DM1 secured FDA approval in 2013 for the treatment of patients with HER2-positive metastatic breast cancer who had previously received trastuzumab and a taxane. This momentous regulatory decision was underpinned by the compelling findings from pivotal clinical trials, namely, the EMILIA trial and the TH3RESA trial [10, 24].

The EMILIA trial (NCT00829166), a randomized, open-label, phase 3 clinical study involving patients afflicted with HER2-positive, unresectable, locally advanced, or metastatic breast cancer who had previously been treated with trastuzumab and a taxane, enrolled 991 patients who were randomly assigned in a 1:1 ratio to receive either T-DM1 or a regimen comprising lapatinib and capecitabine. Results from this trial illuminated that the median progression-free survival (PFS) stood at 9.6 months with T-DM1, whereas it amounted to 6.4 months in the lapatinib plus capecitabine arm. Furthermore, T-DM1 exhibited a superiority in median overall survival (OS) and objective response rate (ORR) over the comparator (29.9 months vs. 25.9 months; 43.6% vs. 30.8%) [27].

The TH3RESA trial (NCT01419197) enrolled 602 patients who were randomly assigned to either the T-DM1 arm (n = 404) or treatment based on the physician's discretion (n = 198). This pivotal investigation revealed a significantly extended median OS (22.7 months vs. 15.8 months with physician's choice-based treatment) and a relatively lower incidence of grade 3 or worse adverse events with T-DM1 (40% vs. 47%) [28].

In the post-approval landscape, clinical exploration of T-DM1 persisted vigorously from 2014 to 2020, with a primary focus on HER2-positive patient populations. Unfortunately, a phase II study in 2016 (NCT02725541) intended for HER2-equivocal patients was withdrawn. Additionally, various treatment modalities and combination therapies involving T-DM1 continued to be subjects of investigation, with further elaboration provided in the subsequent section.

*DS-8201: expanding the landscape of anti-HER2 ADCs:* In the wake of T-DM1, DS-8201 emerged as another prominent anti-HER2 ADC, marked by its rapid development. DS-8201 features a novel topoisomerase I inhibitor payload, DXd, which is connected to trastuzumab by a cleavable linker [10]. Notably, due to its membrane-permeable payload, DS-8201 exhibits an additional impact on HER2-low tumors via a bystander effect [13]. Clinical exploration of DS-8201 commenced in 2017 and has grown to encompass 22 trials as of 2023. These trials can be categorized into 10 studies focusing on DS-8201 as a single agent, 10 studies investigating its combination with other therapies, and 2 studies comparing its efficacy with that of T-DM1.

The pivotal DESTINY-Breast01 trial (NCT03248492) marked the inception of DS-8201 investigations. This single-arm, open-label, multicenter, randomized phase 2 clinical study evaluated the safety and efficacy of DS-8201 as a monotherapy for patients with HER2-positive metastatic breast cancer. Among the 184 patients who had previously received T-DM1, DS-8201 therapy at a dose of 5.4 mg/kg yielded an impressive ORR of 60.9%, a median PFS of 16.4 months, and a median OS of 93.9% at 6 months and 86.2% at 12 months [29]. These compelling results led to the FDA's endorsement of DS-8201 as post-line therapy for HER2-positive breast cancer in 2019.

Subsequently, the DESTINY-Breast02 trial (NCT03523585) in 2018 enrolled 600 patients with HER2-positive unresectable and/or metastatic breast cancer who had previously received T-DM1. This trial compared DS-8201 (n = 406) with treatment selected at the physician's discretion, encompassing either trastuzumab plus capecitabine or lapatinib plus capecitabine (n = 202). DS-8201 achieved a median PFS of 17.8 months versus 6.9 months with physician's choice-based treatment. Importantly, DS-8201 also demonstrated a statistically significant difference in median OS (39.2 months vs. 26.5 months) [30]. These findings validated the efficacy and safety of DS-8201 for advanced HER2-positive breast cancer, offering renewed optimism for clinical management.

Moreover, the groundbreaking DESTINY-Breast03 trial (NCT03529110) marked the first study directly comparing DS-8201 with T-DM1 for advanced HER2-positive breast cancer. In this study, 524 patients were randomly assigned to receive either DS-8201 (n = 261) or T-DM1 (n = 263). DS-8201 showcased a remarkable median PFS of 28.8 months compared to 6.8 months with T-DM1. Importantly, median OS was not reached in either group, and DS-8201 exhibited an impressive ORR of 79% [31]. Based on these findings, the FDA granted approval for DS-8201 as a second-line treatment for advanced HER2-positive breast cancer.

Additionally, the DESTINY-Breast04 trial (NCT03734029) evaluated DS-8201 against physician's chemotherapy choice for metastatic or unresectable HER2-low breast cancer. The results demonstrated a significant improvement in efficacy and safety with DS-8201, highlighting its potential as a therapeutic option. DS-8201 achieved a median PFS of 9.9 months compared to 5.1 months with chemotherapy, with an OS of 23.4 months versus 16.8 months, respectively. Notably, DS-8201 exhibited a lower incidence of grade 3 or higher adverse events [32].

Furthermore, the ongoing DESTINY-Breast05 trial (NCT04622319) is designed to assess DS-8201 versus T-DM1 in patients with HER2-positive primary breast cancer who have residual invasive disease in the breast or axillary lymph nodes and are at a higher risk of recurrence. This ambitious study aims to recruit 1600 patients, with an estimated completion date in 2030 [33].

In the realm of combined therapies, immune checkpoint inhibitors have emerged as prominent partners. DESTINY-Breast06 is a phase 3 trial comparing the efficacy and safety of DS-8201 with chemotherapy for hormone receptor-positive, HER2-low metastatic breast cancer [34]. The most recent trial, initiated in 2023 (NCT05950945), seeks to evaluate the safety and efficacy of DS-8201 in participants with HER2-low unresectable and/or metastatic breast cancer, with outcomes expected to be assessed in 2027.

These trials collectively underscore the transformative impact of DS-8201 in the realm of breast cancer therapeutics, offering renewed hope and expanded treatment options for patients.

*RC48-ADC: an emerging HER2-targeted ADC:* RC48-ADC is a relatively recent addition to the HER2-targeted ADC landscape, consisting of trastuzumab, monomethyl auristatin E (MMAE) as the cytotoxic payload, and a cleavable linker [13]. Initially developed with a focus on urothelial cancers, recent years have seen its potential significance in the context of HER2-low breast cancer.

One noteworthy study, C003 CANCER (NCT03052634), conducted as a phase Ib, open-label, multicenter investigation with three dose cohorts (1.5, 2.0, and 2.5 mg/kg, administered every 2 weeks), commenced in 2016. This trial evaluated the efficacy and safety of RC48-ADC in patients with HER2-positive metastatic breast cancer. Thirty female patients, previously treated with trastuzumab and chemotherapy, were enrolled in the 1.5 mg/kg and 2.0 mg/kg cohorts. Among them, 19 received RC48-ADC, and 16 had undergone three or more prior chemotherapy regimens in the metastatic setting. The study demonstrated a clinical benefit rate (CBR) of 46.7% and an ORR of 26.7% and 46.7% in the 1.5 mg/kg and 2.0 mg/kg cohorts, respectively. Most of the observed adverse events (AEs) were of Grade 1–2 severity, with no AE exceeding Grade 4. These findings underscored the promising potential of RC48-ADC for patients with HER2-positive metastatic breast cancer [35].

Moreover, the most recent study, initiated in July 2023, is comparing RC48-ADC with endocrine therapy for patients with metastatic hormone receptor-positive and HER2-low breast cancer.

*ARX788: a site-specific ADC with tubulin inhibitor payload:* ARX788 represents a site-specific ADC harnessing the potent tubulin inhibitor AS269 as its payload [13]. Its journey in clinical development initiated in 2016 with the NCT02512237 trial, which, regrettably, was terminated due to delayed drug-induced pneumonitis occurring after 4–5 cycles of administration in participants receiving doses at or above 1.3 mg/kg Q3W [36]. Subsequent phase 1 studies in 2018 (NCT03255070) indicated an increasing (ORR ranging from 56 to 63% as doses escalated [26]. Notably, another phase I study showcased a remarkable ORR of 65.5%, a disease control rate (DCR) of 100%, and a median PFS of 17.02 months at a dose of 1.5 mg/kg administered every 3 weeks [37]. Multiple ongoing trials (NCT04829604, NCT04983121, NCT05426486) continue to investigate ARX788 as a monotherapy and in combination with HER2 inhibitors or chemotherapy [38].

*SHR-A1811: an innovative HER2-ADC:* SHR-A1811, an innovative HER2-targeted ADC, embarked on its investigation journey in 2022. At present, limited data is available, with only five trials identified in the available dataset. One notable study combines SHR-A1811 with a PD-1 blocker (NCT05749588).

*Other promising HER2-ADCs*: Several additional HER2-targeted ADCs, including SYD985, have also emerged, offering new avenues of promise. In 2017, a phase 1 study (NCT03262935) compared SYD985 with physician's choice in participants with HER2-positive locally advanced or metastatic breast cancer [39].

The landscape of HER2-targeted ADCs is indeed evolving rapidly, encompassing various agents with the potential to revolutionize the management of HER2-positive and HER2-low breast cancer. Further research and clinical trials are anticipated to shed additional light on their efficacy and safety profiles.

##### TROP2-ADC: expanding beyond HER2

TROP2, initially identified in trophoblast cells, is a transmembrane glycoprotein also found in normal tissues. However, overexpression of TROP2 in epithelial tumors is linked to poor prognosis [24].

*Sacituzumab govitecan (SG): pioneering TROP2-targeted therapy:* Sacituzumab govitecan, often abbreviated as SG, is an ADC that combines a TROP2 antibody (hRS7 IgG1k) with the cytotoxic payload SN-38, utilizing a cleavable linker [10]. It stands as the sole TROP2-ADC approved by the FDA, a milestone achieved following the IMMU-132-01 phase I/II basket trial (NCT01631552) conducted in 2012. This trial was designed to assess the safety and tolerability of IMMU-132 (now known as SG) in patients with advanced epithelial cancers. Among the 108 patients with metastatic triple-negative breast cancer (mTNBC), the study revealed an ORR of 33.3%, a CBR of 45.4%, a durable duration of response (DOR) of 7.7 months, a PFS of 5.5 months, and an OS of 13.0 months. These compelling results led to the accelerated FDA approval of SG for the treatment of adult patients with mTNBC who had previously received at least two prior therapies for metastatic disease [40].

However, after this initial breakthrough, research on SG encountered a subdued phase for the subsequent eight years. It has recently experienced a resurgence, with a focus on combination therapies involving SG from 2016 to 2022, reflecting a renewed interest in exploring its potential.

*Datopotamab deruxtecan (Dato-DXd or DS-1062): advancing TROP2-targeted ADCs: *Datopotamab deruxtecan, often referred to as Dato-DXd or DS-1062, represents a novel TROP2-targeted ADC. It comprises a TROP2 antibody, a topoisomerase 1-targeted payload, and a tetrapeptide-based linker [10]. This promising ADC has already progressed to phase III clinical trials.

The journey for Dato-DXd began with its first clinical trial in 2020 (NCT04644068). This trial aimed to compare the efficacy of AZD5305, a PARP inhibitor, either alone or in combination with other anti-cancer agents, including Dato-DXd and DS-8201, for patients with advanced solid tumors [41]. This early investigation marked the initiation of the TROPION project—a comprehensive clinical development endeavor encompassing a wide range of content to assess the efficacy and safety of Dato-DXd as a single agent and in combination with other anti-cancer therapies for various cancer patients.

Within the scope of TROPION, two pivotal phase III trials have been launched in the context of breast cancer:

TROPION-Breast01 (NCT05104866): This phase III trial is dedicated to evaluating the safety and effectiveness of DS-1062 in patients with inoperable or metastatic HR/HER2 breast cancer who have previously received one or two systems of chemotherapy [42].

TROPION-Breast02 (NCT05374512): Another phase III multicenter study within the TROPION initiative, this trial is designed to assess the efficacy and safety of DS-1062 compared with selected chemotherapy in patients with locally recurrent, inoperable, or metastatic triple-negative breast cancer [43].

These initiatives represent a significant stride in advancing TROP2-targeted ADCs, offering the promise of improved treatment options for patients with various forms of cancer, including breast cancer. As research in this domain continues to progress, further insights into the efficacy and safety of Dato-DXd and related therapies are anticipated.

##### Patritumab deruxtecan (U3-1402 or HER3-DXd): advancing HER3-targeted ADCs

Patritumab deruxtecan, often referred to as U3-1402 or HER3-DXd, represents the sole ADC targeting HER3 currently under investigation. HER3, a member of the HER family proteins, is frequently overexpressed in various malignancies, including breast cancer [10].

U3-1402 is composed of patritumumab and deruxtecan, connected by a tetrapeptide-based cleavable linker [44]. Its clinical journey began in 2016 with the initiation of the first study, focusing on patients with HER3-positive metastatic breast cancer. Subsequently, it was extended to trials involving non-small cell lung cancer (NSCLC) and has reached the phase 3 stage [45]. An upcoming phase 2 trial (NCT05865990) is set to concentrate on patients with metastatic breast cancer or advanced NSCLC with active brain metastases, aiming to further assess its efficacy and safety.

##### MGC018: targeting B7-H3 with an ADC

B7-H3, also known as CD276, belongs to the B7 family and is generally expressed in various human cancers. Its overexpression is strongly associated with cancer proliferation and immune suppression, often leading to poor prognosis [46]. MGC018 is an ADC designed to target B7-H3, consisting of an antibody against B7-H3 and the cytotoxic payload duocarmycin [26]. Clinical trials for MGC018 commenced in 2018 (NCT03729596), with a phase 1/2 study designed to compare the efficacy and safety of MGC018 as a monotherapy and in combination with MGA012, an anti-PD-1 antibody [47].

These developments in the realm of HER3- and B7-H3-targeted ADCs signify significant progress in the field of precision oncology, with the potential to offer improved therapeutic options for patients with breast cancer and other malignancies. As ongoing research unfolds, additional insights into the effectiveness and safety of these ADCs will continue to emerge.

##### FRα: targeting folate receptor alpha with ADCs

Folate Receptor Alpha (FRα), a cell surface receptor responsible for promoting folate intake, is known to be overexpressed in numerous solid tumors, often associated with unfavorable clinical outcomes [26]. MORAb-202 marked a significant development as the first FRα-targeted ADC for BC to enter clinical trials in 2020 (NCT04300556). This ADC comprises anti-FRα farletuzumab and eribulin as payloads. Additionally, two newly developed FRα-ADCs, PRO1184 and AMT-151, are currently undergoing clinical trials. These trials are actively recruiting patients and are aimed at exploring the efficacy of these ADCs for the treatment of advanced solid tumors.

##### gpNMB: targeting glycoprotein NMB with CDX-011

Glycoprotein NMB (gpNMB) is a glycoprotein expressed in various tumor cells and has been associated with promoting cancer progression [26]. CDX-011 is an ADC designed to target gpNMB and was initially researched in 2013. A phase 2 study (NCT01997333) was conducted to evaluate the efficacy and safety of CDX-011 in treating patients with advanced TNBC. This study also involved a comparison with capecitabine, a chemotherapy agent [48].

However, in 2018, two trials (NCT03326258, NCT03473691) focused on investigating CDX-011 for gpNMB-expressing TNBC and unresectable advanced metastatic solid tumors were terminated without providing results. Despite these setbacks, ongoing research efforts in the field of gpNMB-targeted ADCs may hold potential for future developments in the treatment of gpNMB-associated malignancies.

#### ADC as combined therapy: enhancing ADC efficacy

While clinical trials for ADCs have demonstrated significant advancements in breast cancer treatment, the duration of objective response or clinical benefits has remained limited due to the development of drug resistance when ADCs are used as single agents. As a result, the exploration of combined therapies involving ADCs and other anti-tumor agents has become a crucial research direction [49]. Such combination strategies have the potential to enhance the activity of ADCs by various mechanisms, including improving ADC transport to tumors, modulating the expression of target antigens on tumor cells, enhancing the cytotoxicity of payloads, and promoting anti-tumor immune responses [14].

##### ADC plus immunotherapy: harnessing the power of immune checkpoints

Immunotherapy stands as one of the most prevalent combined therapies in ADC clinical trials. Studies have revealed that ADCs may contribute to the effectiveness of immunotherapy through mechanisms such as inducing immunogenic cell death, promoting dendritic cell maturation, increasing T lymphocyte infiltration, and enhancing the expression of immune-regulatory proteins [49]. Notably, the current investigations into ADC combined with immunotherapy predominantly focus on the utilization of PD-1/PD-L1 inhibitors as the immunotherapeutic component.

Based on the data available, a total of 16 trials have explored the combination of ADCs with immunotherapy, with 15 of them specifically targeting PD-1/PD-L1 inhibitors, and one trial involving CD47 as the immunotherapeutic agent.

These endeavors in combining ADCs with immunotherapy represent a promising avenue in the quest to improve the efficacy of breast cancer treatment. By harnessing the synergistic potential of these therapeutic modalities, researchers aim to overcome resistance mechanisms and provide more effective and durable outcomes for patients.

*T-DM1 + Atezolizumab: exploring ADC-immunotherapy combinations:* The KATE2 study (NCT02924883) represents a significant milestone as the first phase 2 trial to investigate the combination of an ADC with immunotherapy. In this randomized, double-blind, placebo-controlled study, 202 patients with HER2-positive locally advanced or metastatic breast cancer were assigned to receive either T-DM1 plus atezolizumab (n = 133) or T-DM1 plus placebo (n = 69) [50]. The median PFS was 8.2 months for the T-DM1 plus atezolizumab group, slightly longer than the 6.8 months observed in the T-DM1 plus placebo group. However, this difference did not reach clinical significance. Notably, serious adverse events occurred in 33% of patients in the T-DM1 plus atezolizumab group and 19% in the T-DM1 plus placebo group. Nevertheless, a prespecified exploratory analysis suggested a potential PFS benefit in the subgroup of patients with PD-L1-positive tumors. This intriguing finding prompted further investigation, leading to the initiation of a phase 3 trial known as KATE3 (NCT04740918) in 2021.

The KATE3 trial enrolled 350 patients with centrally-determined HER2-positive and PD-L1-positive unresectable locally advanced or metastatic BC. These patients had previously received trastuzumab (with or without pertuzumab) and taxane-based therapy. Similar to KATE2, the trial used a 1:1 allocation, with one group receiving T-DM1 plus atezolizumab and the other receiving T-DM1 plus placebo [51]. The results of this study are anticipated to provide further insights into the potential benefits of combining T-DM1 with atezolizumab and are expected to be available in 2024.

*T-DM1 + Pembrolizumab: safety and tolerability assessment:* In 2017, a phase 1b study (NCT03032107) was conducted to assess the safety and determine the appropriate dose for the combination of T-DM1 and pembrolizumab in metastatic HER2-positive breast cancer. The primary endpoint of this study was safety and tolerability. Secondary endpoints included ORR and PFS [52]. The study results showed that the combination of T-DM1 and pembrolizumab was indeed safe and well-tolerated. However, the evidence for improved efficacy, as measured by ORR (20%) and median PFS (9.6 months), was limited. While the safety profile was favorable, the clinical benefits of this combination therapy lacked robust clinical evidence at this stage.

These trials illustrate the ongoing efforts to explore the potential benefits of combining ADCs with immunotherapy in the treatment of breast cancer. The results of larger-scale trials, such as KATE3, are eagerly anticipated to provide more comprehensive insights into the effectiveness of these combinations.

*DS-8201 + Nivolumab: combination therapy for HER2-expressing tumors:* In a phase 1b study designated DS8201-A-U105 (NCT03523572), researchers explored the potential of combining DS-8201 with Nivolumab for patients with HER2-expressing advanced/metastatic tumors, with a focus on two cohorts dedicated to BC. The results of this study provided valuable insights into the effectiveness of this combination in different subsets of patients [53].

The study found that in HER2-positive patients, the combination demonstrated an impressive ORR of 65.6%, indicating a substantial proportion of patients experiencing a positive response to treatment. In HER2-low patients, the ORR was 50.0%. The median PFS for the HER2-positive group was 11.6 months, while in the HER2-low group, it was 7.0 months.

It's worth noting that when comparing these results with the PFS of DS-8201 as a single agent in the DESTINY-Breast01 trial (16.4 months), the combination therapy did not appear to provide a clear advantage. However, it's important to consider several factors that might contribute to these differences. These factors include variations in patient demographics, baseline characteristics, sample sizes, and the specific patient populations in each trial. Therefore, the results do not necessarily dismiss the clinical potential of combining an ADC with immunotherapy, and further investigation is warranted.

*DS-8201/Dato-DXd + Durvalumab: ongoing research in BEGONIA:* In the BEGONIA trial (NCT03742102), researchers are exploring the combination of DS-8201 and Dato-DXd with durvalumab in various cohorts, including those with different levels of PD-L1 expression. This study aims to evaluate the responses and efficacy of these combination therapies in different patient populations [54].

The preliminary results indicate that responses have been observed in both PD-L1-low and PD-L1-high patients, suggesting that these combinations may have activity in a broad range of individuals. However, it's important to note that the trial is ongoing, with estimated completion in 2024. As more data becomes available from this study, it will provide further insights into the potential benefits of combining ADCs with immunotherapy in the treatment of breast cancer and other tumor types.

*Dato-DXd + Durvalumab: TROPION-Breast03:* The TROPION-Breast03 trial (NCT05629585) is intended to assess the efficacy and safety of Dato-DXd, a TROP2-ADC, with or without a PD-1 inhibitor. This study will aim to evaluate whether the combination of Dato-DXd with a PD-1 inhibitor results in improved outcomes for patients with breast cancer.

*SG + Atezolizumab: ASPRIA study:* The ASPRIA trial (NCT04434040), initiated in 2020, is an open-label, single-arm phase 2 study designed to evaluate the combination therapy of SG and atezolizumab in patients with TNBC who have residual invasive disease following neoadjuvant therapy. This study aims to assess the potential benefits of combining SG, an ADC, with atezolizumab, an immune checkpoint inhibitor, in this specific patient population.

In this trial, 40 patients are expected to be enrolled and will receive a total of 6 cycles of adjuvant therapy composed of the combination of SG and atezolizumab. The primary endpoint of the study is the rate of undetectable circulating tumor cell-free DNA after completing the 6 cycles of therapy. The ASPRIA trial is projected to be completed in 2023 [55].

*SG + Pembrolizumab: ASCCENT-04 and ASCENT-05 trials:* The ASCCENT-04 (NCT05382286) and ASCENT-05 (NCT05633654) trials, initiated in 2022, represent two global, open-label, randomized, phase 3 studies that aim to investigate the potential benefits of combining SG with pembrolizumab in patients with mTNBC. These studies specifically focus on patients with PD-L1-positive tumors [56, 57].

In these trials, patients will be randomly assigned in a 1:1 ratio to receive either SG plus pembrolizumab or treatment selected by their physician plus pembrolizumab. The goal of these studies is to assess whether the combination of SG and pembrolizumab results in improved efficacy compared to standard treatments in this patient population [56, 57].

*DS-8201 + ALX148: investigating the CD47 inhibitor:* An intriguing phase 1 trial (NCT05868226), initiated in 2022, is designed to evaluate the efficacy of DS-8201 alone or in combination with ALX148. ALX148 is an inhibitor that targets the novel immune checkpoint CD47. This trial aims to explore the potential of combining DS-8201, an ADC, with ALX148 to enhance its therapeutic effects, possibly by modulating immune responses through the CD47 pathway.

The trial is currently in the recruiting stage, and as it progresses, it may provide valuable insights into the synergy between DS-8201 and CD47 inhibition as a potential treatment approach for breast cancer and other malignancies.

TROPION-Breast03: assessing Dato-DXd with a PD-1 inhibitor: The outcomes of these trials, expected to be obtained in the future, will provide further insights into the potential benefits of combining ADCs with various immunotherapies, including PD-1 inhibitors and CD47 inhibitors, in the treatment of breast cancer.

##### ADC plus chemotherapy

Chemotherapy is also a common choice for combination with ADC. But it does have some limitation considering the payload itself is also chemical agent. The cell cycle, administration time, modulation of surface antigens and overlapping toxicity should be taken into consideration [49]. Trials for ADC combining chemotherapy were not much as immunotherapy, and it is still at the exploring period.

*T-DM1 + Taxane: combination trials:* Combining T-DM1 with taxanes, such as lapatinib and abraxane, has been explored in clinical trials for patients with metastatic HER2-positive breast cancer. These trials aimed to assess the potential benefits of combining T-DM1, an ADC, with taxane-based chemotherapy regimens.

NCT02073916: This phase Ib trial, initiated in 2013, investigated the combination of T-DM1 with lapatinib and abraxane for a total of 4 cycles. Among the 14 patients evaluable for response, the trial reported that 42.9% experienced grade 3 or higher toxicity at the maximum tolerated dose (MTD) [58].

NCT02073487: This trial, initiated in 2014, also explored the combination of T-DM1 with lapatinib and abraxane in patients with metastatic HER2-positive breast cancer. It aimed to evaluate the safety and efficacy of this combination regimen in this patient population [59].

*T-DM1 + Anthracycline: THELMA trial:* The THELMA trial (NCT02562378), initiated with the goal of determining the MTD, examined the combination of T-DM1 with non-pegylated liposomal doxorubicin (NPLD) in patients with metastatic breast cancer. This study focused on patients who had previously received taxanes and trastuzumab-based therapy.

In this trial, the median PFS was reported as 7.2 months, with an ORR of 40% and a CBR of 46.7%. However, it's important to note that every patient in the study experienced at least one adverse event, suggesting that toxicity remains a significant limitation for this treatment approach [60].

##### ADC plus anti-HER2

Because of the improved therapeutic index of ADCs and their activity on selective tumor populations, they are promising partners for targeted agents [49]. HER2 inhibitors such as pertuzumab are most popular to be adopted for ADC combination study.

*T-DM1 + Pertuzumab:* Combination therapy involving the ADC T-DM1 and the HER2 inhibitor pertuzumab has been explored in several phase 3 clinical trials for the treatment of HER2-positive breast cancer. While the trials did not demonstrate significant improvements in certain endpoints, they provided valuable insights into the safety and efficacy of this combination approach.

KAITLIN (NCT01966471): The KAITLIN trial was a phase 3, randomized, open-label study involving 1,846 patients with HER2-positive primary invasive breast cancer. The results of the trial showed no significant difference between the arms in terms of invasive disease-free survival (IDFS) event risk, indicating that the combination of T-DM1 and pertuzumab did not significantly improve this endpoint [61].

KRISTINE (NCT02131064): The KRISTINE trial compared the efficacy of T-DM1 plus pertuzumab with docetaxel, carboplatin, trastuzumab plus pertuzumab for HER2-positive stage 2–3 breast cancer. While T-DM1 plus pertuzumab led to a lower pathological complete response (pCR) rate compared to the chemotherapy regimen (44.4% vs. 55.7%), it was associated with fewer Grade 3 or higher adverse events, suggesting a favorable safety profile for this combination [62].

MARIANNE (NCT01120184): The MARIANNE trial enrolled 1,095 patients with HER2-positive advanced breast cancer and randomized them to receive T-DM1 plus pertuzumab, T-DM1 plus placebo, or trastuzumab plus a taxane. The median OS was found to be similar across the treatment groups [63]. While the trial did not demonstrate a significant OS benefit for the combination of T-DM1 and pertuzumab, it also indicated a low rate of adverse events associated with this combination therapy.

These trials, despite their negative results in terms of certain endpoints, highlight the importance of assessing the safety and efficacy of ADC-HER2 inhibitor combinations in the treatment of HER2-positive breast cancer. The favorable safety profile observed in some of these trials may have implications for future treatment strategies.

*T-DM1 + Tucatinib:* The combination of T-DM1 with tucatinib has shown promising results in the treatment of advanced or metastatic HER2-positive breast cancer, as demonstrated in the HER2CLIMB-02 trial (NCT03975647):

HER2CLIMB-02 (NCT03975647): This phase 3 trial, initiated in 2019, aimed to evaluate whether the combination of T-DM1 and tucatinib is more effective than T-DM1 alone in patients with advanced or metastatic HER2-positive breast cancer. The trial showed clinically meaningful survival benefits for the combination group, with a median duration of OS of 24.7 months compared to 19.2 months in the placebo group. Median duration of PFS was also longer in the combination group (7.6 months vs. 4.9 months). Importantly, the combination therapy was well-tolerated, with a low rate of adverse events [64].

Additionally, another phase 3 trial (NCT04457596) initiated in 2021 is assessing the efficacy of T-DM1 and tucatinib in preventing recurrence in patients with high-risk HER2-positive breast cancer. This trial is ongoing and is expected to provide further insights into the potential benefits of this combination in a specific patient population [65].

*T-DM1 + Neratinib:* The FB-10 study (NCT02236000) is a phase Ib/II trial designed to assess the dose-limiting effects and efficacy of combining T-DM1 with neratinib in patients with metastatic HER2-positive breast cancer:

FB-10 study (NCT02236000): This trial involved 27 patients treated with various dose levels of neratinib in combination with T-DM1 for metastatic HER2-positive breast cancer. Among the 19 patients evaluable for response, the study aimed to determine the safety and efficacy of this combination therapy [66].

*DS-8201 + Tucatinib:* In the context of HER2-positive unresectable locally advanced or metastatic breast cancer, the HER2CLIMB-04 trial (NCT04539938) is exploring the safety and antitumor activity of combining DS-8201 with tucatinib:

HER2CLIMB-04 (NCT04539938): This single-arm, open-label phase 2 trial, initiated in 2020, is evaluating the combination of DS-8201 with tucatinib in patients with HER2-positive unresectable locally advanced or metastatic breast cancer. All participants will receive both DS-8201 and tucatinib. The primary completion of this trial is expected in 2024 [67].

##### ADC plus PIK3Ca inhibitor

The combination of ADCs with PIK3Ca inhibitors is an area of research interest in breast cancer treatment. Here are some notable trials exploring this combination:

*T-DM1 + BYL719 (PIK3 inhibitor):* In a phase I trial initiated in 2014 (NCT02038010), researchers investigated the combination of T-DM1 with BYL719, an oral PIK3 inhibitor. The trial involved 14 patients who were evaluable for response. The results showed an ORR of 43%, which was higher than the ORR observed in patients who had previously received treatment with T-DM1 and had disease progression (30%). This suggests the potential efficacy of combining T-DM1 with a PIK3 inhibitor in the treatment of HER2-positive breast cancer [68].

*SG + Alpelisib (PI3Kα inhibitor):* In 2022, a phase 1 trial named ASSET (NCT05143229) was initiated to investigate the safety and efficacy of combining SG with alpelisib, a PI3Kα inhibitor. The trial enrolled 18 patients with metastatic or locally recurrent HER2-negative breast cancer. This study aims to assess the feasibility and potential benefits of the SG and alpelisib combination in patients with this subtype of breast cancer. The results of this trial will provide valuable insights into the use of PI3K inhibitors in combination with ADCs for breast cancer treatment.

##### ADC + CDK4/6 inhibitor

The combination of ADCs with CDK4/6 (cyclin-dependent kinase 4/6) inhibitors is an area of research interest in breast cancer treatment. Here are some notable trials exploring this combination:

*T-DM1 + Palbociclib*: In a phase 1b study initiated in 2014 (NCT01976169), researchers investigated the combination of T-DM1 with Palbociclib (PD-0332991) in patients with recurrent or metastatic HER2-positive breast cancer. The trial aimed to determine the appropriate dose of T-DM1 in combination with Palbociclib. The results showed an objective ORR of 33% and a median PFS of 6 months. Hematologic toxicity was the most common Grade 3 toxicity observed in more than 10% of patients. This trial suggested a resensitizing effect of CDK4/6 inhibitors in HER2-resistant breast cancer, providing valuable insights into potential combination therapies [69]. Another phase 2 study (NCT03530696) initiated in 2018 further explores whether the combination of T-DM1 and Palbociclib can improve PFS in metastatic HER2-positive breast cancer.

*T-DM1 + Ribociclib:* In 2016, a phase 1b study (NCT02657343) investigated the safety and sought to define the appropriate dose of T-DM1 in combination with ribociclib in advanced or metastatic HER2-positive breast cancer [70]. However, the results showed an ORR of only 16.7% and a median PFS of 10.4 months. It's worth noting that CDK4/6 inhibitors like ribociclib block the process of tumor cells entering the S/M period of the cell cycle, which may affect the efficacy of T-DM1. A phase 2 trial (NCT04351230) initiated in 2020 aimed to study the efficacy of T-DM1 with or without Abemaciclib, another CDK4/6 inhibitor, but was unfortunately withdrawn with no results.

##### ADC plus endocrine therapy

TRIO-US B-12 TALENT (NCT04553770) is a phase 2 neoadjuvant trial conducted in 2020 to investigate DS-8201 with or without anastrozole for HER2-low, HR-positive early-stage breast cancer [71]. In this trial, patients were randomized 1:1 to receive six cycles of DS-8201 alone or in combination with anastrozole. The results are expected to be reported in 2025, as estimated.

DESTINY-Breast 08 (NCT04556773) is an ongoing phase 1b trial focused on exploring the safety, tolerability, pharmacokinetics, and anti-tumor activity of DS-8201 in combination with other therapies, including anastrozole and fulvestrant [72].

## Current status and future prospects in antibody–drug conjugates for breast cancer treatment

Tracing the journey from Paul Ehrlich's conception of a 'magic bullet' in the early 1900s to the FDA's approval of T-DM1 in 2013, ADCs for BC represent a field that has evolved substantially over a century [14]. Despite persistent challenges, the landscape has experienced transformative improvements. Presently, quantities of ADC therapies have been successfully developed, thereby positively affecting the lives of thousands of patients afflicted with cancer. The approval of 14 distinct ADC compounds, along with their noteworthy clinical outcomes, has increasingly solidified interest in this relatively nascent yet extraordinarily intricate domain. Given the continued investment in research and development, the future of ADCs is anticipated to unveil further promising advancements in the targeted treatment of neoplastic diseases.

### Current research landscape and challenges in ADC therapeutics for breast cancer

#### Evolution of ADC components

ADCs can be taxonomically classified into three generations based on their constituent components and methodologies. The first generation principally encompasses conventional chemotherapeutic agents, mouse-derived antibodies, and non-cleavable linkers, which are plagued by numerous limitations including unstable linkers, antibody aggregation, and heterogeneous agents. The second-generation ADCs leverage IgG1 isotype monoclonal antibodies (mAbs), cytotoxic payloads with increased potency, and optimized linkers, collectively enhancing the therapeutic index of these conjugates. The third generation features precisely characterized drug-to-antibody ratios (DARs) of either 2 or 4 and deploys fully humanized antibodies to minimize immunogenicity [73]. Collectively, advancements in ADCs are highly contingent upon the iterative optimization of these components. However, due to the large molecular weight of ADC compared with other cytotoxic drugs, the efficiency of drug penetration remains a limitation [73]. Current research foci include the identification of novel targets, introduction of polyclonal antibodies, and the design of payloads with greater tumor-specific toxicity and enhanced stability. As for linkers, the emphasis is on developing ones that degrade selectively within neoplastic tissues, sparing normal cells.

#### Synergistic approaches: combination with other therapeutics

While certain ADCs like T-DM1 and DS-8201 have secured regulatory approval for the treatment of BC, the therapeutic durability of these monotherapies remains circumscribed, largely due to the emergence of drug resistance. Currently discovered resistance mechanisms developed by tumors include downregulation of antigens, modification of intracellular pathway, and resistance to the payloads [73]. Consequently, investigations into the co-administration of ADCs with other therapeutic methods have gained attention. Numerous clinical trials have focused on combining ADCs with immunotherapeutic agents, predominantly with inhibitors of the PD-1/PD-L1 axis. Additionally, efforts to amalgamate ADCs with standard chemotherapeutics are ongoing; however, overlapping toxicities present considerable challenges. Molecularly targeted agents, particularly HER2 inhibitors, have also been integrated into combination strategies. Employing ADCs in conjunction with endocrine therapies represents a nascent yet burgeoning approach. The linchpin for successful combination therapies resides in augmenting therapeutic efficacy while maintaining an acceptable tolerability profile.

#### Mitigating toxicological concerns

Owing to its considerable molecular weight, ADC administration is commonly conducted via intravenous injection, a route that poses a potential risk of significant dermal toxicity. Besides, the most severe side effect of ADC proved to be hematotoxicity, which, along with hepatotoxicity and gastrointestinal reaction, is largely resulted from premature release of the payloads [73]. Ideally, ADCs should remain intact within the circulatory system, releasing their cytotoxic payloads exclusively in the proximity of tumor cells. Inadvertent payload release in systemic circulation can induce off-target toxicity. Strategies to mitigate these adverse effects are multifaceted, encompassing dose optimization, component refinement, incorporation of pharmacogenomic mapping, and early toxicity screening [74].

#### Identification of novel biomarkers

The therapeutic efficacy of ADCs is intrinsically linked to the specificity of antibody-antigen interactions, particularly those antigens that are either overexpressed or uniquely expressed on target cells. Consequently, the discovery of new biomarkers serving as targeted antigens is crucial for advancing ADC innovation [75]. Current research indicates that HER3 and FRα represent promising targets that are actively under investigation.

### Future directions in ADC therapeutics for breast cancer

Notwithstanding the extant challenges, the realm of ADCs holds considerable promise, with specific emphasis on emerging innovations such as bispecific ADCs and peptide-drug conjugates (PDCs) [73].

#### Bispecific and dual-targeting ADCs

Advancements in the field of bispecific antibody technologies are poised to revolutionize ADC development by enhancing both antibody internalization and tumor specificity. Similarly, the emergence of dual-targeting ADCs and dual-payload ADCs offers a compelling strategy to mitigate the incidence of drug resistance.

#### Peptide-drug conjugates (PDCs)

An alternative frontier in ADC development involves the utilization of truncated peptide sequences as vehicles for payload conjugation, rather than employing conventional antibodies. This approach aims to diminish molecular weight, thereby facilitating improved penetration into tumors that are anatomically challenging to access. Nevertheless, issues associated with rapid plasma clearance warrant further investigation.

#### ADCs targeting mutant proteins

In contrast to their wild-type counterparts, mutant proteins typically exhibit elevated levels of ubiquitination, which facilitates their internalization and subsequent degradation. Harnessing ADCs to target these mutant proteins could potentially augment tumor specificity, particularly in neoplasms characterized by oncogenic mutations.

#### Dual-payload strategies

Developing dual-specificity ADCs, which can target two different antigens simultaneously, is another innovative approach. This strategy can enhance therapeutic precision, reduce the likelihood of drug resistance, and potentially address multiple cancer cell populations within a tumor, providing a more comprehensive treatment.

#### Non-internalizing ADCs

The limited dissemination of antibodies within solid tumors, attributable to antigenic barriers, has been a long-standing obstacle in the field. Development of non-internalizing ADCs, which facilitate extracellular payload release, represents an innovative approach that could potentially enhance therapeutic outcomes.

## Data Availability

The data collected are available as open data via ClinicalTrials.gov: https://www.clinicaltrials.gov and the WHO International Clinical Trials Registry Platform (ICTRP): https://trialsearch.who.int.
